# Peptidylarginine deiminase in *Porphyromonas gingivalis*-derived outer membrane vesicles exacerbates metabolic dysfunction-associated steatotic liver disease through the NPAS2/CYP4A10 pathway

**DOI:** 10.1186/s12951-026-04523-x

**Published:** 2026-05-09

**Authors:** Yanqing Liu, Xiaomeng Xue, Zhaorong Li, Ziming Ge, Muzhou Jiang, Jingbo Liu, Yaping Pan, Li Lin

**Affiliations:** 1 Department of Periodontology, School and Hospital of Stomatology, China Medical University, Shenyang, Liaoning China; 2 Department of Oromaxillofacial–Head and Neck Surgery, Department of Oral and Maxillofacial Surgery, School and Hospital of Stomatology, China Medical University, Shenyang, Liaoning China; 3 Liaoning Provincial Key Laboratory of Oral Disease, Shenyang, Liaoning China

**Keywords:** periodontitis, *P. gingivalis* OMVs, peptidylarginine deiminase, MASLD, NPAS2

## Abstract

This study investigated the relationship between periodontitis and metabolic dysfunction-associated steatotic liver disease (MASLD), with a specific focus on the role of *Porphyromonas gingivalis* peptidylarginine deiminase (PPAD) carried by *P. gingivalis*-derived outer membrane vesicles (OMVs) in hepatic lipid metabolism. Clinical analyses revealed a positive correlation between periodontal disease severity and the fatty liver index (OR = 6.18, 95% CI: 2.36–16.13), a predictor of MASLD. In an obese mouse model of periodontitis, *P. gingivalis*-induced periodontitis accelerated MASLD progression, promoted hepatic lipid accumulation and increased inflammation. Since *P. gingivalis* OMVs had the advantage of traveling through the blood to the liver, we found that they demonstrated liver-specific accumulation, impairing fatty acid oxidation and increasing lipid deposition in hepatocytes, when injected into MASLD model mice via the tail vein. The critical role of PPAD was confirmed using the *P. gingivalis*^*Δppad*^ and *P. gingivalis*^*ppad−oe*^ strains, from which OMVs were isolated. The results of the in vivo and in vitro experiments indicated that PPAD-enriched OMVs mediated hepatic metabolic dysregulation via suppression of the circadian regulator NPAS2 and downstream inhibition of CYP4A10 expression, revealing a previously unrecognized PPAD–liver communication pathway. These findings highlight the urgent need for effective PPAD inhibitors and a deeper understanding of the interactions of PPAD with host proteins implicated in systemic diseases, underscoring the broader health impacts of periodontal disease.

**Graphical Abstract:**

**Figure:**
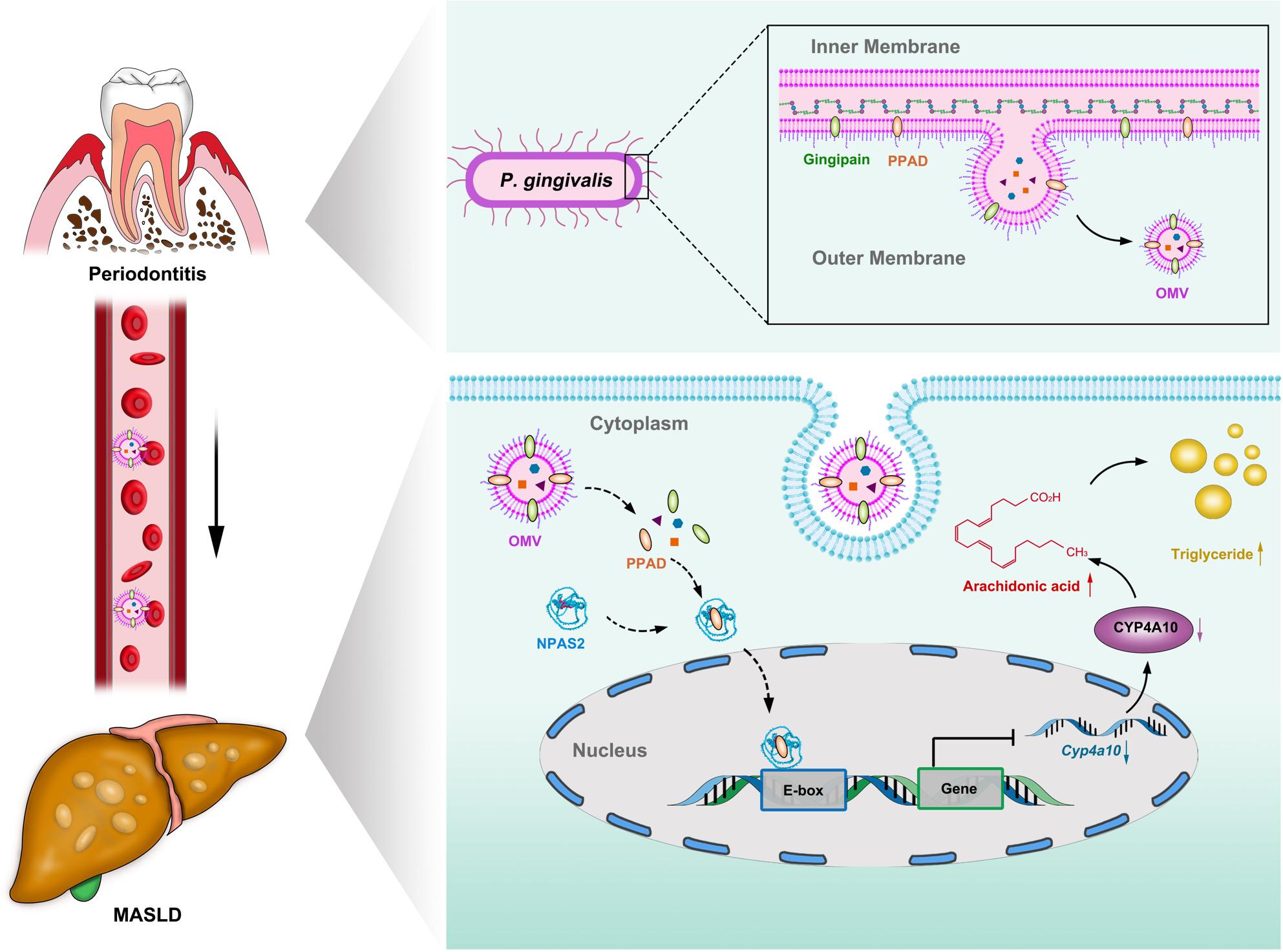
On the basis of our findings, we propose a working model in which PPAD carried within *P. gingivalis* OMVs may disrupt hepatic lipid metabolism via an interaction with NPAS2 and downregulation of CYP4A10

**Supplementary Information:**

The online version contains supplementary material available at 10.1186/s12951-026-04523-x.

## Introduction

Periodontitis is a common oral condition affecting 20–50% of the global population [1]. It is associated with various systemic health issues, including cardiovascular diseases, Alzheimer’s disease, and liver disorders such as fatty liver, liver fibrosis, cirrhosis, and liver cancer [2–6]. Of particular interest is its connection to metabolic dysfunction-associated steatotic liver disease (MASLD), formerly termed nonalcoholic fatty liver disease (NAFLD). MASLD, linked to metabolic syndrome, progresses from simple steatosis to a severe inflammatory stage known as metabolic dysfunction-associated steatohepatitis (MASH), which can lead to cirrhosis and hepatocellular carcinoma [7]. Accumulating evidence highlights the bidirectional relationship between periodontitis and MASLD [8]. Epidemiological studies—both cross-sectional and longitudinal—have indicated that periodontitis is a risk factor for MASLD [9–11].

*Porphyromonas gingivalis (P. gingivalis*), a key periodontal pathogen, plays an important role in the interplay between periodontitis and MASLD. In a study involving 200 MASLD patients who underwent liver biopsies and measurements of the serum *P. gingivalis* antibody titre, a positive correlation was found between liver fibrosis severity and *P. gingivalis* antibody titres [12]. Evidence from clinical, basic, and immunological studies suggests that *P. gingivalis* infection promotes lipid accumulation, amplifies immune responses, induces insulin resistance, and accelerates MASLD progression [13, 14].

Outer membrane vesicles (OMVs) shed by *P. gingivalis* are critical virulence factors capable of penetrating host tissues and activating proinflammatory pathways [15]. Recent genetic, proteomic, and morphological studies have revealed that OMVs encapsulate bacterial outer membrane components and serve as carriers of virulence factors. These vesicles induce inflammatory responses, damage host cells, and facilitate the delivery of pathogenic agents [16, 17]. The vesicular membrane structure of OMVs protects their contents from degradation, enabling their long-distance transport and amplifying their harmful effects compared to those of the bacteria themselves [18]. Studies in mouse models have indicated that OMVs containing gingipain proteases can reach the liver, alter glucose metabolism, and contribute to diabetes mellitus [19]. However, their potential role in hepatic lipid metabolism remains unclear. One important *P. gingivalis* virulence factor is peptidylarginine deiminase (PPAD), an enzyme identified in 1999 [20] that modifies proteins and promotes inflammation by increasing neutrophil survival and cytokine secretion [21]. PPAD also interacts with gingipain R to produce citrullinated proteins, which are implicated in noncommunicable diseases such as rheumatoid arthritis, atherosclerosis, and Alzheimer’s disease [22]. However, whether PPAD carried within OMVs contributes to MASLD progression has yet to be determined.

To evaluate the effect of periodontitis on MASLD, we validated the relationship between periodontitis and MASLD using clinical samples and in vivo animal models. We further investigated the role and possible molecular mechanism of OMVs derived from *P. gingivalis*, an important periodontal pathogen, in hepatic lipid metabolism and the progression of MASLD. These findings provide new insights into the mechanisms linking periodontitis with systemic diseases, advancing the understanding of integrated health management.

## Results

### Clinical analysis demonstrated a positive correlation between the severity of periodontal disease and MASLD

The presence of MASLD was determined using two validated indices, the fatty liver index (FLI) and the hepatic steatosis index (HSI), with the presence of MASLD defined by a FLI > 60 and a HSI > 36 [11]. Periodontal disease severity was assessed using established criteria [23], with inclusion and exclusion criteria detailed in Appendix Table 1. A total of 92 participants were enrolled in the study and were categorized into two groups: the NO/Stage I–II periodontitis group (*n* = 46) and the Stage III–IV periodontitis group (*n* = 46). The baseline participant characteristics are summarized in Appendix Table 2.

**Fig. 1:**
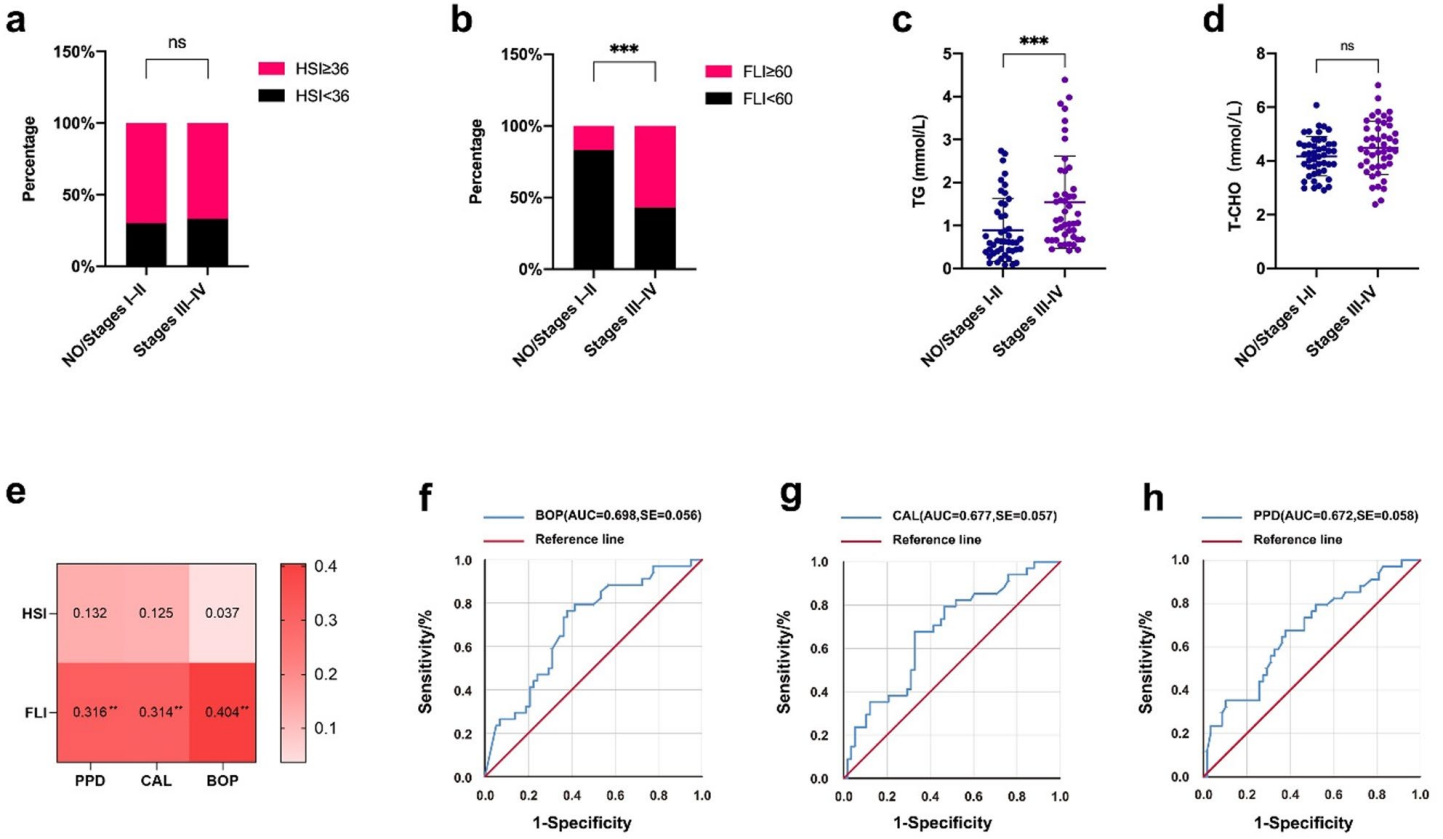
Clinical studies indicated a positive correlation between the severity of periodontal disease and MASLD.**a**,** b** The percentages of HSI and FLI in various periodontitis patients were compared using chi-square test. **c** Comparison of TG content in blood of different patients with periodontitis. Statistical significance was determined using the Mann-Whitney test. Data were expressed as the mean ± SD. **d** Comparison of T-CHO content in blood of different patients with periodontitis. Statistical significance was determined using the T test. Data were expressed as the mean ± SD. **e** Spearman correlation analysis of periodontitis correlation index with HIS and FLI. Red means positive correlation. **f**,** g**,** h** The ROC curve of PPD, CAL and BOP for predicting HSI and FLI. *n* = 46 both in the NO/Stages I–II periodontitis and Stages III–IV periodontitis group. **P < 0.01, ***P < 0.001. TG, triglyceride; T-CHO, total cholesterol; HSI, hepatic steatosis index; FLI, fatty liver index

The results revealed a significant positive correlation of stage III–IV periodontitis with a FLI > 60 (OR = 6.18, 95% CI: 2.36–16.13; *P* < 0.001) but no significant correlation with a HSI > 36 (*P* > 0.05; Fig. 1a, b). The serum triglyceride (TG) concentrations were significantly elevated in the Stage III–IV periodontitis group compared with the NO/Stage I–II periodontitis group (*P* < 0.001), whereas the total cholesterol (T-CHO) concentrations were not significantly different between the groups (*P* > 0.05; Fig. 1c, d). Univariate analyses and logistic regression models (Appendix Tables 3 and 4) were used to examine the relationships between the FLI and HSI values and periodontal indicators. Univariate analysis showed associations between bleeding on probing (BOP), clinical attachment loss (CAL), and periodontal probing depth (PPD) and the FLI. However, after adjusting for confounding factors such as age, sex, BMI, smoking status, alcohol use status, hypertension status, and diabetes status, these associations were no longer significant. Spearman correlation analysis revealed that PPD (*r* = 0.316), CAL (*r* = 0.314), and BOP (*r* = 0.404) were positively correlated with the FLI (*P* < 0.01). In contrast, PPD (*r* = 0.132), CAL (*r* = 0.125), and BOP (*r* = 0.037) were weakly but nonsignificantly correlated with the HSI (*P* > 0.05; Fig. 1e).

A receiver operating characteristic (ROC) curve was used to evaluate the predictive efficacy of clinical periodontal indicators for the FLI. The area under the curve (AUC) for BOP was 0.698 (95% CI: 0.589–0.807; *P* = 0.002), indicating a strong predictive ability. When the BOP threshold was 31.5%, the sensitivity and specificity for predicting the FLI were 76.5% and 62.1%, respectively. Similarly, the AUC for CAL was 0.677 (95% CI: 0.565–0.789; *P* = 0.005), indicating good predictive efficiency. At a threshold of 3.18 mm, the sensitivity and specificity of CAL were 67.6% and 67.2%, respectively. The AUC for PPD was 0.672 (95% CI: 0.559–0.786; *P* = 0.006), also indicating high predictive efficiency. When the PPD threshold was 2.885 mm, the sensitivity and specificity for predicting the FLI were 67.6% and 62.1%, respectively (Fig. 1f–h).

### Oral infection with *P. gingivalis* exacerbated the progression of MASLD and promoted lipid accumulation in the livers of obese mice

**Fig. 2:**
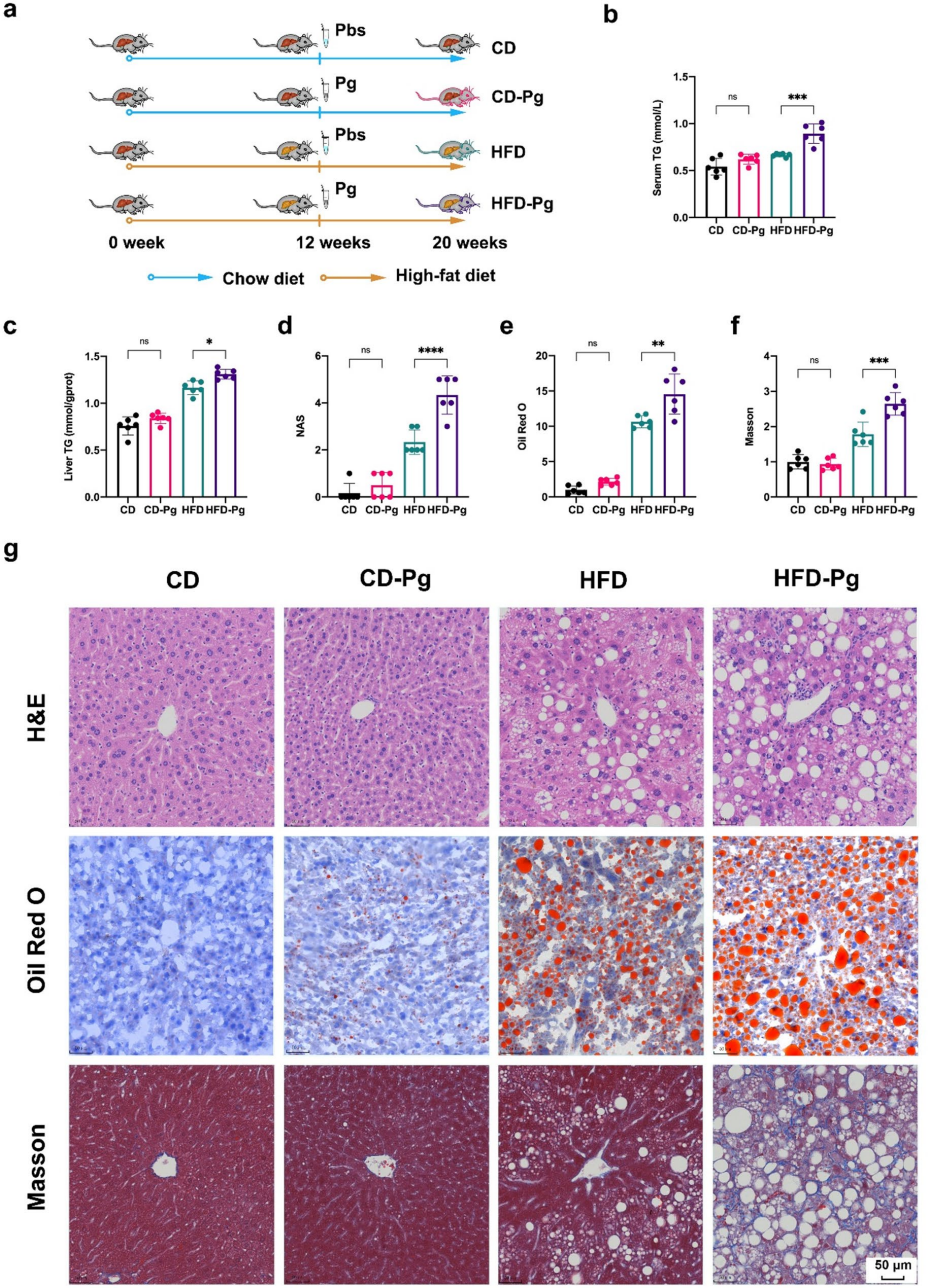
Oral infection with *P. gingivalis* aggravated the progression of MASLD and promoted lipid accumulation in the liver of mice. **a** Schematic of the animal experimental design in the four groups (*n* = 6 in each group). **b** Serum levels of TG in each group. **c** TG content per gram of liver from the indicated groups of mice. **d** NAFLD activity score (NAS) in each group. **e** Quantification of Oil Red O positive area per field in each group. **f** Quantitative analysis of liver fibrosis in each group. **g** Representative photomicrographs for H&E, Oil Red O and Masson staining of the liver sections from mice (scale bars = 50 μm). *P < 0.05, **P < 0.01, ***P < 0.001, ****P < 0.0001. MASLD, metabolic dysfunction-associated steatotic liver disease; TG, triglyceride

To investigate the impact of *P. gingivalis* infection on MASLD progression, a mouse model was established. The mice were fed either a high-fat diet (HFD group, *n* = 6) for 12 weeks or a standard chow diet (CD group, *n* = 6) as controls. *P. gingivalis* infection (10⁹ CFU per dose, three times per week) was subsequently induced by application to the mandibular molars for eight weeks in both the CD-Pg and HFD-Pg groups (Fig. 2a). MASLD progression was evaluated using the NAFLD activity score (NAS) [24].

The serum and hepatic TG levels were significantly greater in the HFD-Pg group than in the HFD group (*P* < 0.05), whereas no significant differences were detected between the CD and CD-Pg groups (*P* > 0.05; Fig. 2b, c). The NAS was notably higher in the HFD-Pg group than in the HFD group (*P* < 0.0001; Fig. 2d). Histological analysis revealed more severe hepatic steatosis, lobular inflammation, hepatocyte ballooning, and fibrosis in the HFD-Pg group than in the HFD group (Fig. 2e–g). The results of Oil Red O staining confirmed the greater lipid accumulation in the HFD-Pg group (*P* < 0.01). Compared with the CD group, the CD-Pg group exhibited increased inflammatory infiltration but no significant difference in hepatic steatosis or ballooning. Similarly, the Oil Red O staining results were not significantly different between the CD and CD-Pg groups. These results suggest that *P. gingivalis*-induced periodontitis may increase hepatic fat accumulation and exacerbate MASLD progression.

### *P. gingivalis* OMVs can be transferred to the liver both in vivo and in vitro

To investigate the role of *P. gingivalis* OMVs, an important pathogenic factor of *P. gingivalis*, in hepatic lipid metabolism and the progression of MASLD, we isolated *P. gingivalis* OMVs and conducted in vivo and in vitro tracking experiments.

**Fig. 3:**
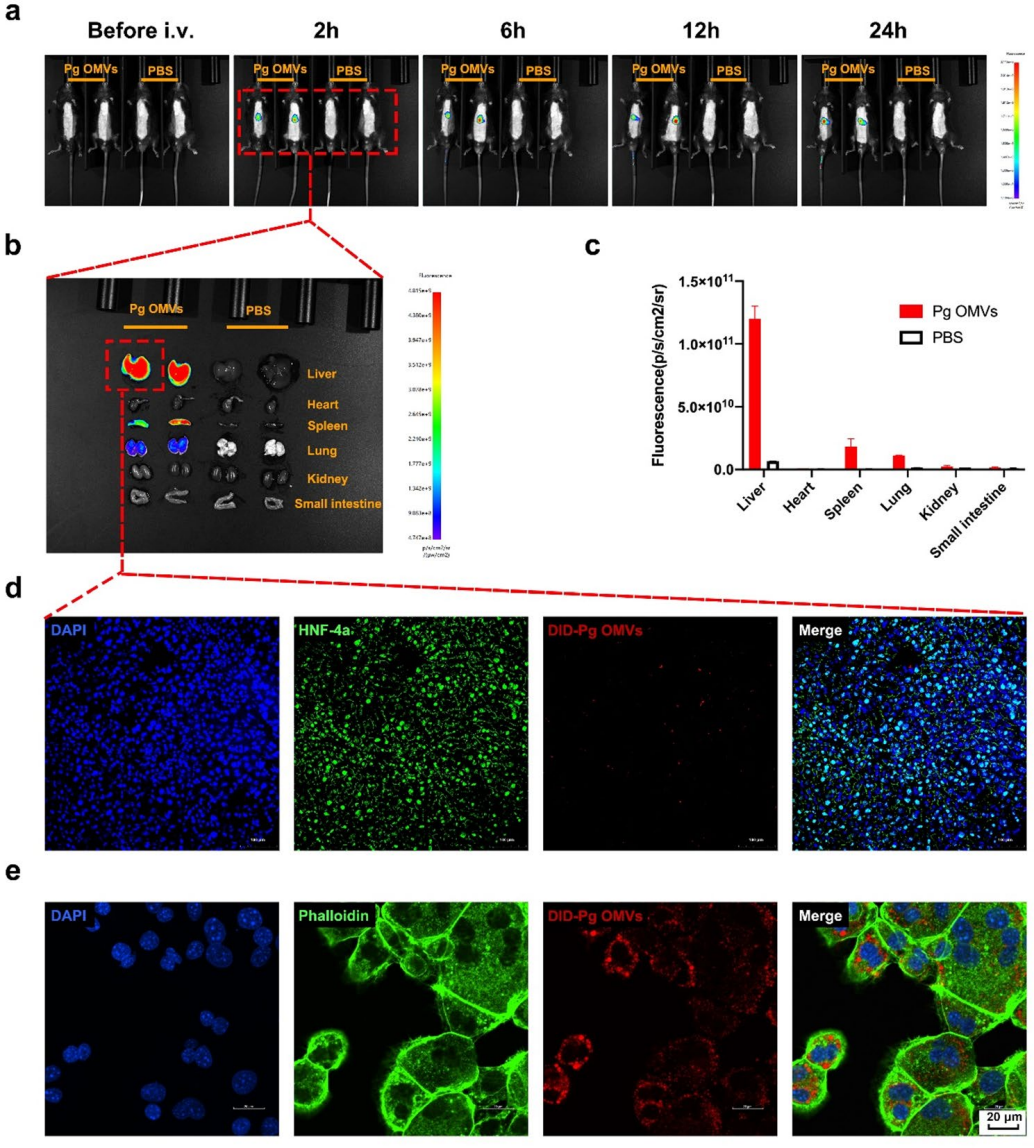
*P. gingivalis* OMVs can be transferred to the liver cells both in vivo and in vitro. **a** DiD-labeled *P. gingivalis* OMVs were injected into C57BL/6J mice through the tail vein and their distribution was recorded at 2 h, 6 h, 12 h, and 24 h after injection using IVIS. The colour scale represents radiant efficiency. **b**,** c** The fluorescence distribution and histogram of liver, heart, spleen, lung, kidney and small intestine. **d** The DiD-labeled *P. gingivalis* OMVs in mouse liver was observed by immunofluorescence. Scale bar = 100 μm. **e** The DiD-labeled *P. gingivalis* OMVs in AML12 cells was observed using confocal microscopy. Scale bar = 20 μm. OMVs, outer membrane vesicles

Transmission electron microscopy (TEM) analysis of *P. gingivalis* outer membrane vesicles (OMVs) revealed their characteristic cup-like morphology and bilayer vesicle structure (Fig. S1a). Nanoparticle tracking analysis (NTA) revealed an OMV diameter of 136.0 nm, consistent with that of typical gram-negative bacterial OMVs (Fig. S1b). For in vivo analysis, DiD-labelled OMVs (DiD-Pg OMVs) were injected into mice via the tail vein (Fig. S1c). IVIS imaging at 2, 6, 12, and 24 h post-injection revealed liver-specific accumulation, which peaked at 12 h (Fig. 3a). Strong fluorescence was detected in the excised liver, spleen, and lungs, with the liver exhibiting the highest fluorescence intensity (Fig. 3b, c). The results of confocal laser microscopy further confirmed the liver localization of the OMVs (Fig. 3d). In the in vitro experiment, AML12 cells exposed to DiD-Pg OMVs for 6 h internalized the vesicles, as confirmed by immunofluorescence analysis (Fig. 3e).

### *P. gingivalis* OMVs can exacerbate the progression of MASLD by affecting fat metabolism-related processes in the liver

**Fig. 4:**
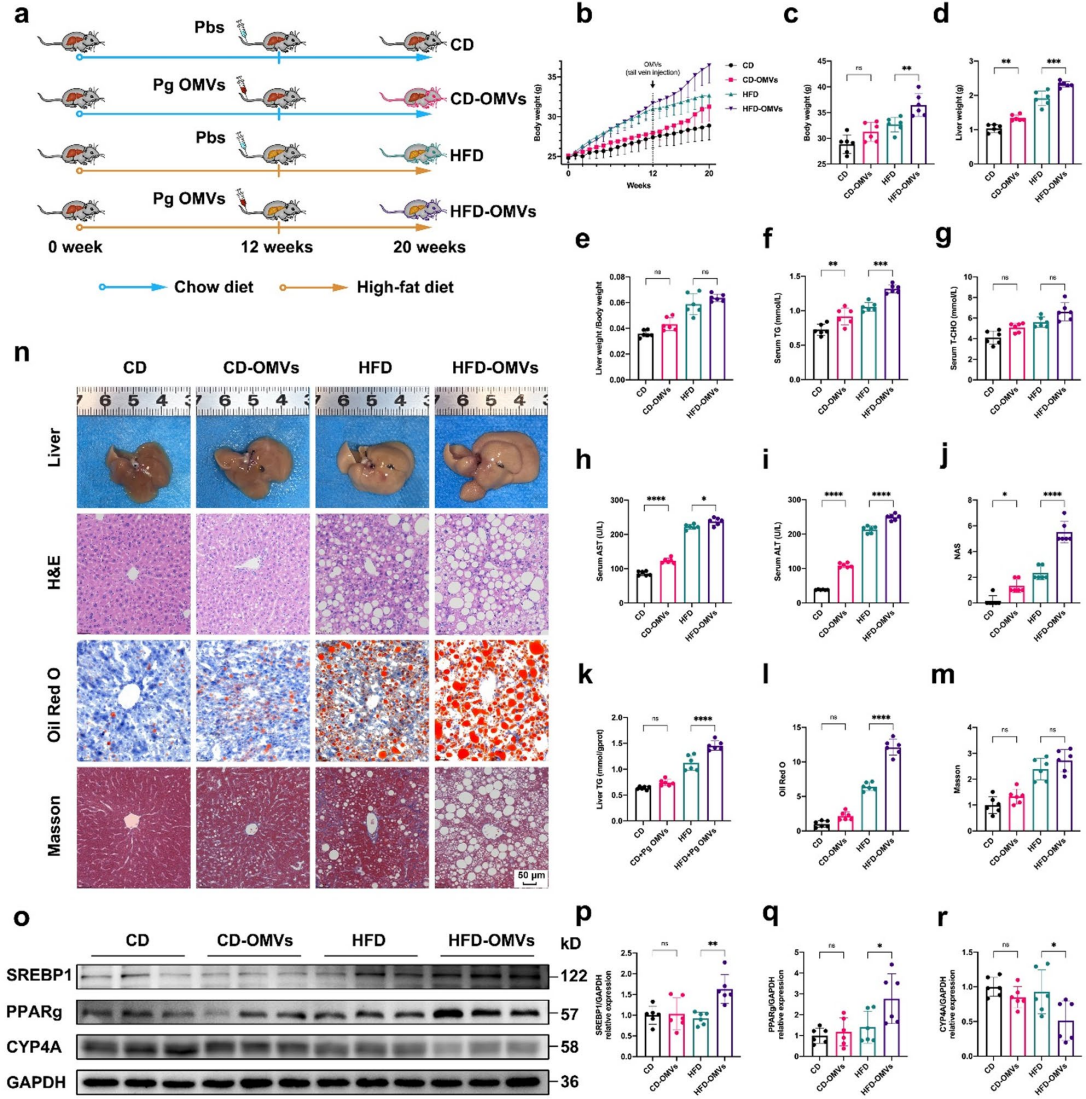
*P. gingivalis* OMVs can aggravate the progression of MASLD by affecting fat metabolism processes in the liver. **a** Schematic of the animal experimental design in the four groups (*n* = 6 in each group). **b** Body weight change of mice in each group. In CD-OMVs and HFD-OMVs groups, *P. gingivalis* OMVs was injected into the tail vein of mice at 12 weeks. **c-e** Final body weight, liver weight, liver weight/body weight of mice in each group. **f-i** The levels of TG, T-CHO, AST and ALT in the serum of mice in each group. **j** NAS score in each group. **k** Liver TG content of mice in each group. **l** Quantification of Oil Red O positive area in each group. **m** Quantitative analysis of liver fibrosis in each group. **n** Representative photos of liver and representative images of H&E, Oil Red O staining and Masson staining of liver sections. **o-r** Analysis of SREBP1, PPARg and CYP4A expression in the livers of mice by western blot. Scale bar = 50 μm. *P < 0.05, **P < 0.01, ***P < 0.001, ****P < 0.0001. MASLD, metabolic dysfunction-associated steatotic liver disease; OMVs, outer membrane vesicles; CD, chow diet; HFD, high-fat diet; TG, triglyceride; T-CHO, total cholesterol; AST, Aspartate Aminotransferase; ALT, Alanine Aminotransferase; NAS, NAFLD activity score

To evaluate the effects of *P. gingivalis* OMVs on MASLD, additional experiments were performed (Fig. 4). The body and liver weights of the mice in the HFD-OMVs group were significantly higher than those of the mice in the HFD group (*P* < 0.01). However, no significant difference was observed in the liver-to-body weight ratio (*P* > 0.05; Fig. 4b–e). The serum TG concentrations were higher in the HFD-OMVs group than in the HFD group (*P* < 0.001), while the T-CHO concentrations remained comparable (*P* > 0.05; Fig. 4f, g). Notably, the serum Aspartate Aminotransferase (AST) and Alanine Aminotransferase (ALT) concentrations were significantly elevated in both the HFD-OMVs and CD-OMVs groups, indicating liver injury (*P* < 0.05; Fig. 4h, i). The NASs were also higher in the OMV-treated groups than in the control groups (*P* < 0.05; Fig. 4j).

Histological analysis of the liver revealed pronounced steatosis, lobular inflammation, and hepatocyte ballooning in the HFD-OMVs group compared with the HFD group, although the increase in fibrosis in the HFD-OMVs group was not significant. Compared with the CD group, the CD-OMVs group exhibited increased inflammatory cell infiltration and mild hepatocyte ballooning (Fig. 4n). Compared with that in the HFD group, the hepatic TG content in the HFD-OMVs group was significantly elevated (*P* < 0.0001; Fig. 4k). The results of Oil Red O staining confirmed this observation, with a higher fluorescence intensity in the HFD-OMVs group than in the HFD group (*P* < 0.0001). However, no statistically significant difference in the fluorescence intensity was observed between the CD-OMVs and CD groups (*P* > 0.05; Fig. 4l). In contrast to the HFD-Pg group, which exhibited significantly increased collagen deposition compared to that in the HFD control group, the HFD-OMVs group exhibited visible collagen deposition, but the increase compared with that in the HFD control group was not statistically significant (Fig. 4m).

Gene expression analysis of fat metabolism-related enzymes revealed significant upregulation of lipogenic genes (*Fasn*, *Scd1*, and *Pparg*) and downregulation of fatty acid oxidation-related genes (*Cpt1a* and *Cyp4a*) in the HFD-OMVs group compared with the HFD group (Fig. S2). The results of Western blotting confirmed increases in the protein expression of SREBP1 and PPARγ, along with a decrease in CYP4A expression (Fig. 4o–r).

### *P. gingivalis* OMVs affect lipid metabolism in the mouse liver by inhibiting the mRNA expression of *Npas2*

**Fig. 5:**
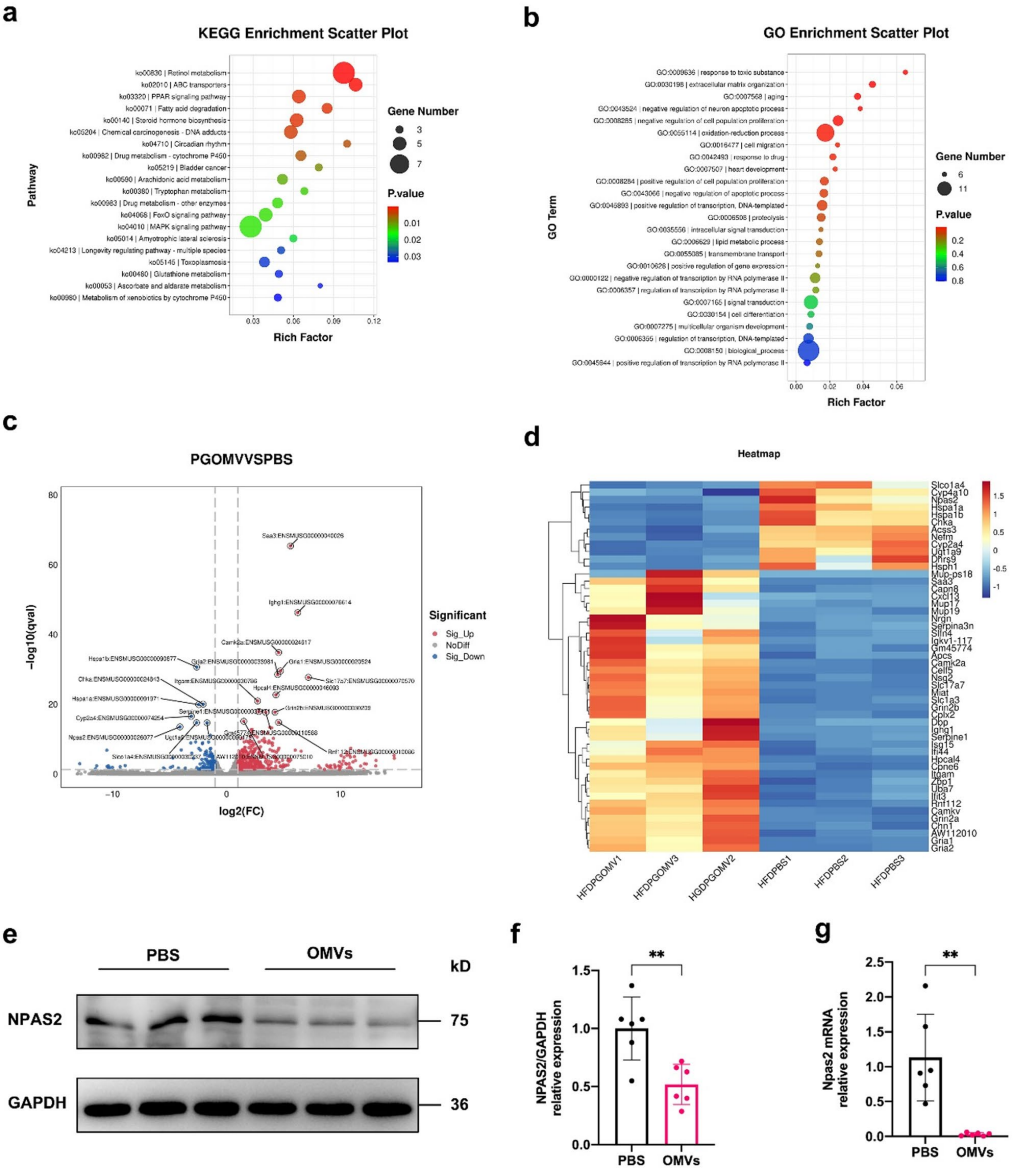
*P. gingivalis* OMVs affects lipid metabolism in mouse liver by inhibiting the expression of *Npas2.* **a** Enrichment analysis results of KEGG pathway. **b** Enrichment analysis results of GO term. **c** The volcano map of top 20 differential expression genes. **d** The heat map of differential gene expression. **e-g** Analysis of *Npas2* expression in the livers of mice by qRT-PCR and western blot. **P < 0.01. OMVs, outer membrane vesicles

To investigate the mechanisms by which *P. gingivalis* OMVs affect hepatic fat metabolism, transcriptome sequencing was performed on liver tissues from the HFD-OMVs and HFD control groups. A p value cut-off of 0.05 and a fold change (FC) threshold of 2 were used. A total of 630 differentially expressed genes (DEGs) were identified, with 455 upregulated and 175 downregulated (Fig. S3a). Kyoto Encyclopedia of Genes and Genomes (KEGG) pathway enrichment analysis revealed that the pathways enriched in the differentially expressed genes included not only pathways related to circadian rhythm, cytochrome P450 metabolism, MAPK signalling and other signalling types but also PPAR signalling, fatty acid degradation, arachidonic acid metabolism and other processes (Fig. 5a). Gene Ontology (GO) functional enrichment analysis revealed enrichment of the differentially expressed genes mainly in terms related to lipid metabolism, oxidation–reduction processes, ageing, proteolysis, transcriptional regulation, and multicellular biological development (Fig. 5b). Visualizations, including a volcano plot of the top 20 DEGs and a differential expression heatmap, are provided in Fig. 5c and d. Additional data visualizations, such as a Venn diagram, GO and KEGG enrichment bar plots, and an ES line diagram for arachidonic acid epoxygenase activity, are shown in Fig. S3b–e. Validation analysis of the top 20 DEGs using qRT‒PCR revealed expression trends consistent with the sequencing results for *Npas2*, *Cyp2a4*, *Itgam*, and *Serpine1* (Fig. S3f–h). On the basis of a literature review, *Npas2* (log2 FC = -4.19, *P* < 0.01) was selected for further validation in mouse liver tissue by qRT‒PCR and Western blotting (Fig. 5e–g).

### *P. gingivalis* OMVs downregulate NPAS2 expression and disrupt lipid metabolism in hepatocytes

**Fig. 6:**
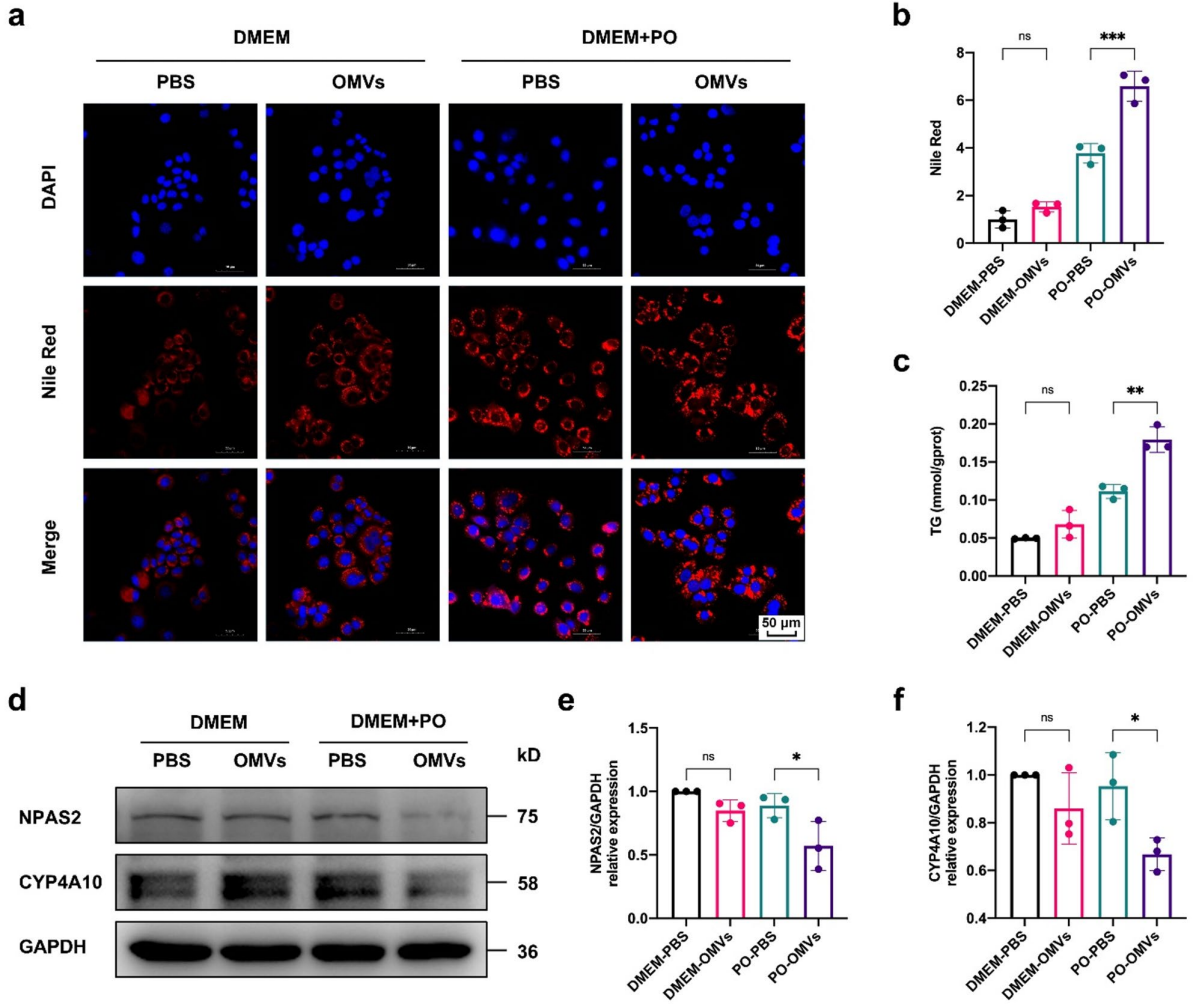
AML12 cells were exposed to *P. gingivalis* OMVs led to the increase in lipid droplet accumulation. **a** AML12 cells, with or without PO solution, were treated with *P. gingivalis* OMVs or PBS and stained with Nile Red. Scale bar = 50 μm. **b** The histogram of Nile Red staining statistics. **c** TG content of AML12 cells in each group as described in **a. d-f** Analysis of NPAS2 and CYP4A10 expression in the AML12 cells by western blot. *n* = 3 in each group, *P < 0.05, **P < 0.01, ***P < 0.001. OMVs, outer membrane vesicles; PO solution, palmitic acid and oleic acid solution, TG, triglyceride

To evaluate the role of NPAS2 in the metabolic effects of *P. gingivalis* OMVs, we established an in vitro MASLD model using AML12 cells treated with a solution containing palmitic acid (250 µM) and oleic acid (500 µM) (PO) for 24 h. The optimal OMV concentration and treatment duration were determined using a cell counting kit-8 (CCK-8) assay and measurements of TG accumulation (Fig. S4a, b). AML12 cells were then incubated with 2 µg/ml *P. gingivalis* OMVs for 6 h. This treatment significantly increased the intracellular TG levels and lipid droplet fluorescence, as visualized by Nile red staining (*P* < 0.01; Fig. 6a–c). Furthermore, NPAS2 expression was markedly reduced (Fig. 6d, e), and the expression of CYP4A10, a key enzyme in oxidative lipid metabolism, was significantly decreased. The downregulation of *Cyp4a10* expression was confirmed by RNA sequencing (log_2_ FC = -1.37, *P* < 0.05) and validated by qRT‒PCR (Fig. S3i).

### *P.gingivalis* OMVs affect lipid metabolism in hepatocytes through the NPAS2/CYP4A10 pathway

**Fig. 7:**
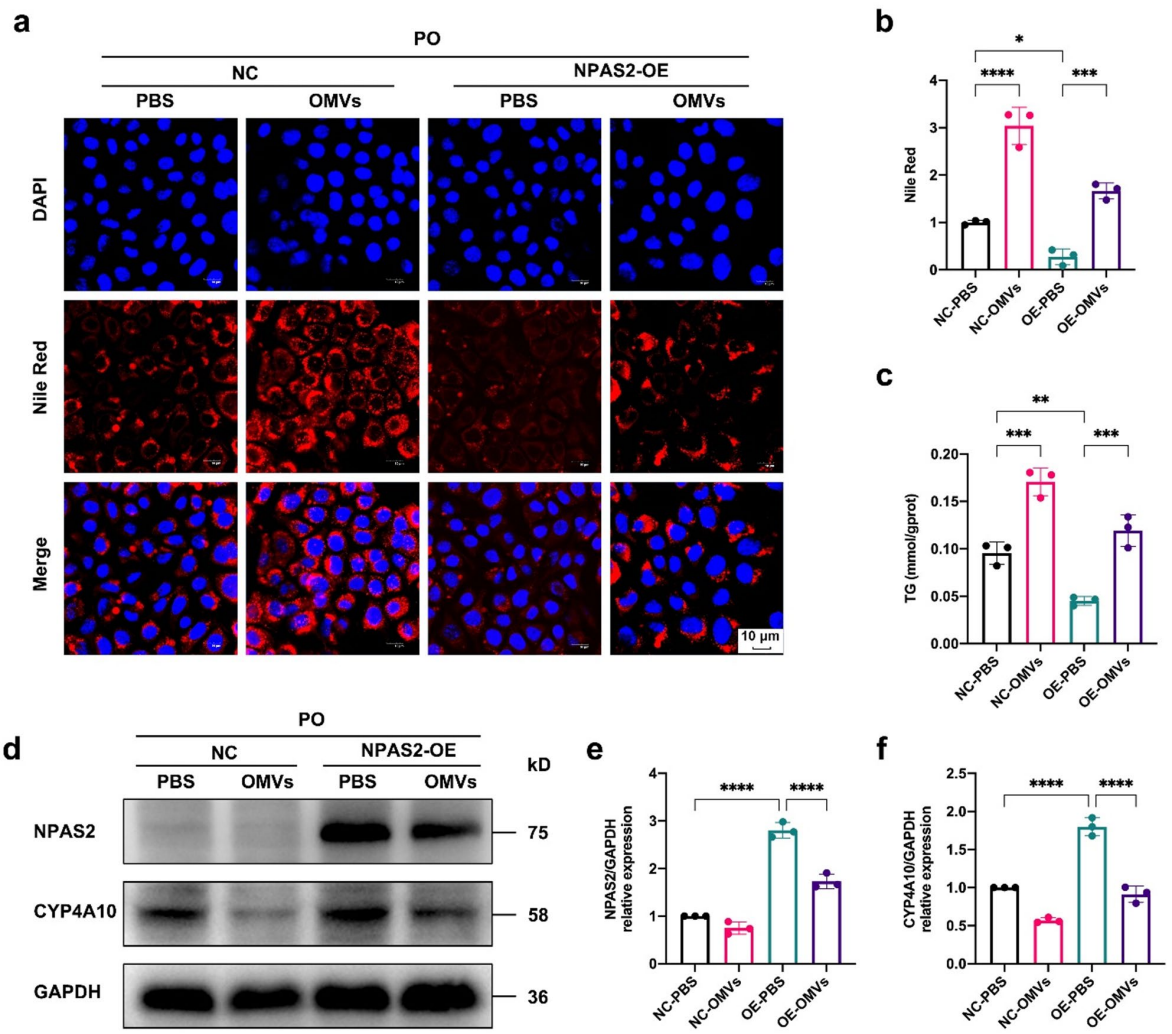
*P. gingivalis* OMVs affects the metabolism of fat in hepatocytes through the NPAS2/CYP4A10 pathway. **a** NPAS2–overexpressing plasmid and NC plasmid transfected AML12 cells were treated with *P. gingivalis* OMVs or PBS and stained with Nile Red. Scale bar = 50 μm. **b** The histogram of Nile Red staining in each group. **c** TG content of AML12 cells in each group as described in **a. d**-**f** Analysis of NPAS2 and CYP4A10 expression in each group cells as described in **a** by western blot. *n* = 3 in each group, *P < 0.05, **P < 0.01, ***P < 0.001, ****P < 0.0001. OMVs, outer membrane vesicles; TG, triglyceride

To investigate the role of NPAS2 in hepatocyte-mediated MASLD pathogenesis, we generated NPAS2-overexpressing (NPAS2-OE) AML12 cells and incubated them with PO solution for 24 h. Successful transfection and selection were indicated by the widespread ZsGreen fluorescence, and NPAS2 overexpression was subsequently validated by Western blot analysis of whole-cell lysates (Fig. S5). Lipid accumulation was assessed using Nile red staining in transfected AML12 cells treated with *P. gingivalis* OMVs or PBS (Fig. 7a). Compared with the NC-PBS group, the OE-PBS group exhibited significantly lower lipid accumulation and TG levels (*P* < 0.05; Fig. 7b, c), indicating that NPAS2 is critical for lipid metabolism in AML12 cells. Interestingly, compared with the OE-PBS group, the OE-OMV group displayed increased lipid accumulation and TG levels (*P* < 0.001), suggesting that fat accumulation induced by *P. gingivalis* OMVs is partially regulated by NPAS2, with additional mechanisms potentially involved (Fig. 7b, c). Western blot analysis revealed that CYP4A10 protein expression was significantly elevated in the OE-PBS group compared with the NC-PBS group (*P* < 0.0001) (Fig. 7d–f), demonstrating a positive regulatory relationship between NPAS2 and CYP4A10. However, compared with that in the OE-PBS group, CYP4A10 expression in the OE-OMV group was notably reduced, suggesting that *P. gingivalis* OMVs affect hepatocyte lipid metabolism via the NPAS2/CYP4A10 pathway (Fig. 7d-f).

### PPAD carried by *P. gingivalis* OMVs affects lipid metabolism in the liver through the NPAS2/CYP4A10 pathway and exacerbates the progression of MASLD

In this study, we also explored whether PPAD carried by *P. gingivalis* OMVs contributes to MASLD pathogenesis. OMVs were isolated from the *P. gingivalis*^*Δppad*^ strain, and their bilayer vesicle-like structure (126.2 nm in diameter) was confirmed using TEM and NTA (Fig. S6a). The effects of these OMVs on lipid metabolism were evaluated in both in vivo and in vitro models.

**Fig. 8:**
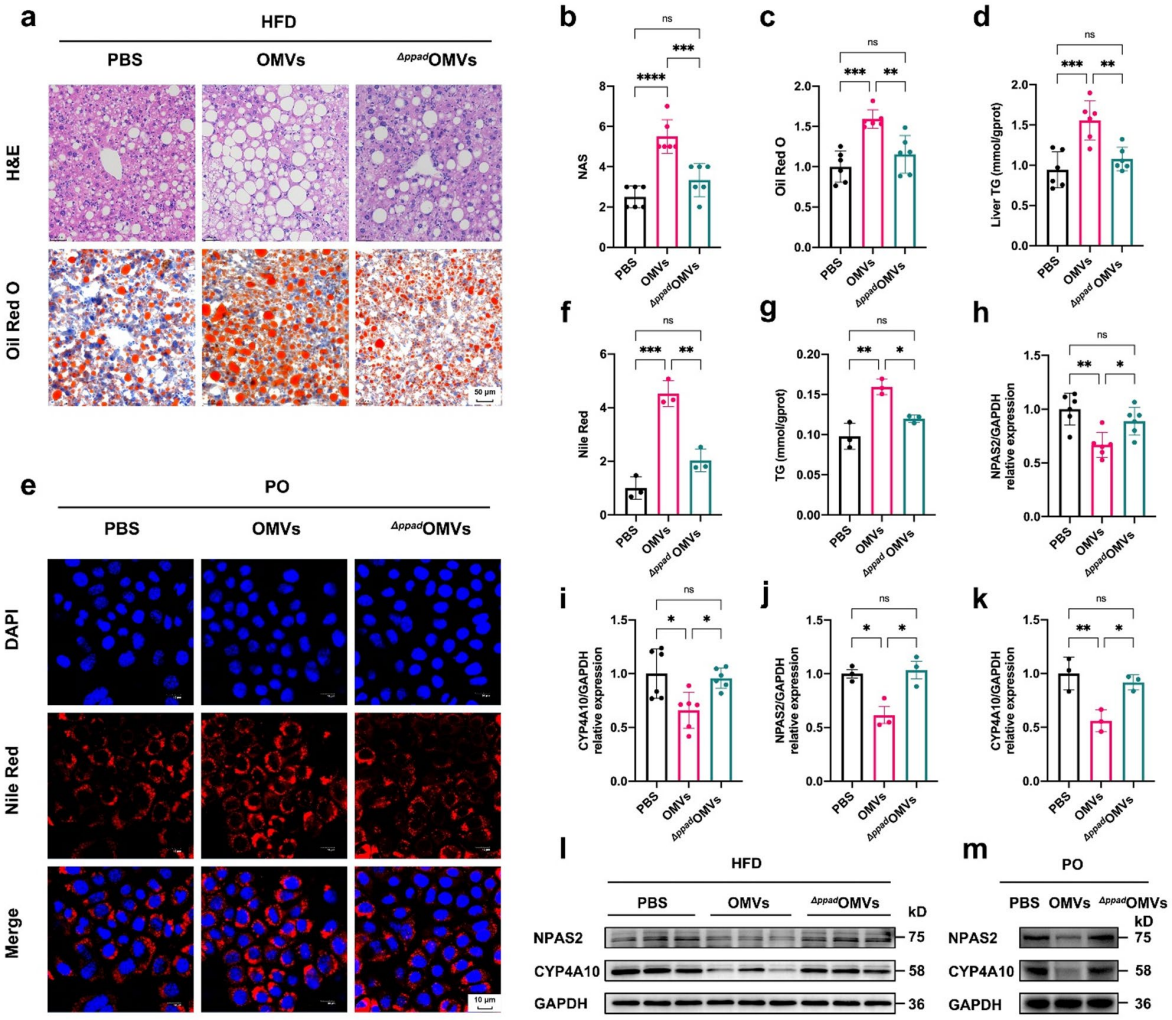
The PPAD carried by *P. gingivalis* OMVs affects lipid metabolism in the liver through the NPAS2/CYP4A10 pathway and aggravates the progression of MASLD. **a** Representative photomicrograph for H&E, Oil Red O staining of the liver sections from mice of PBS group, *P. gingivalis* OMVs group and *P. gingivalisΔppad* OMVs group. (Scale bars = 50 μm). **b** NAS score in each group. **c** Quantification of Oil Red O positive area in each group. **d** Liver TG content of mice in each group. **e** AML12 cells cultured with PO solution for 24 h were treated with *P. gingivalis* OMVs, *P. gingivalisΔppad* OMVs and pbs and then stained with Nile Red. Scale bar = 10 μm. **f** The histogram of Nile Red staining statistics. **g** TG content of AML12 cells in each group. **h**,** i**,** l** Analysis of NPAS2 and CYP4A10 expression in mice in each group by western blot. **j**,** k**,** m** Analysis of NPAS2 and CYP4A10 expression in AML12 cells in each group by western blot. *n* = 3 in each group. Statistical significance was determined by one-way ANOVA followed by Tukey‘s multiple comparisons test. *P < 0.05, **P < 0.01, ***P < 0.001, ****P < 0.0001. OMVs, outer membrane vesicles; PO solution, palmitic acid and oleic acid solution, TG, triglyceride. NAS, NAFLD activity score

For the in vivo studies, 18 mice were fed a high-fat diet for 12 weeks and randomly divided into three groups to receive tail vein injections of *P. gingivalis* OMVs (1 µg/µl, 5 µl, three times weekly for 8 weeks; OMV group), *P. gingivalis*^*Δppad*^ OMVs (1 µg/µl, 5 µl, three times weekly for 8 weeks; ^*Δppad*^OMV group), or PBS (5 µl, three times weekly for 8 weeks; PBS group). The ^*Δppad*^OMV group exhibited significantly lower NASs, lipid accumulation, and TG concentrations than the OMV group (*P* < 0.01) and no significant differences compared to the PBS group (Fig. 8a–d).

For the in vitro studies, AML12 cells were cultured in PO solution for 24 h and stimulated with *P. gingivalis* OMVs (2 µg/ml; OMV group), *P. gingivalis*^*Δppad*^ OMVs (2 µg/ml; ^*Δppad*^OMV group), or PBS (2 µl/ml; PBS group). Six hours post-stimulation, the results mirrored those observed in vivo, with statistically significant differences between the ^*Δppad*^OMV and OMV groups (*P* < 0.05; Fig. 8e–g). Western blot analysis of mouse liver tissues and AML12 cells revealed no significant differences in NPAS2 or CYP4A10 protein expression between the ^*Δppad*^OMV and PBS groups. In contrast, these proteins were downregulated in the OMV group (Fig. 8h–m). These findings suggest that PPAD carried in *P. gingivalis* OMVs modulates hepatic lipid metabolism through the NPAS2/CYP4A10 pathway, contributing to MASLD progression.

### The binding of PPAD to NPAS2 may be mediated through the interaction of Asp291 of PPAD with Arg231 of Npas2

**Fig. 9:**
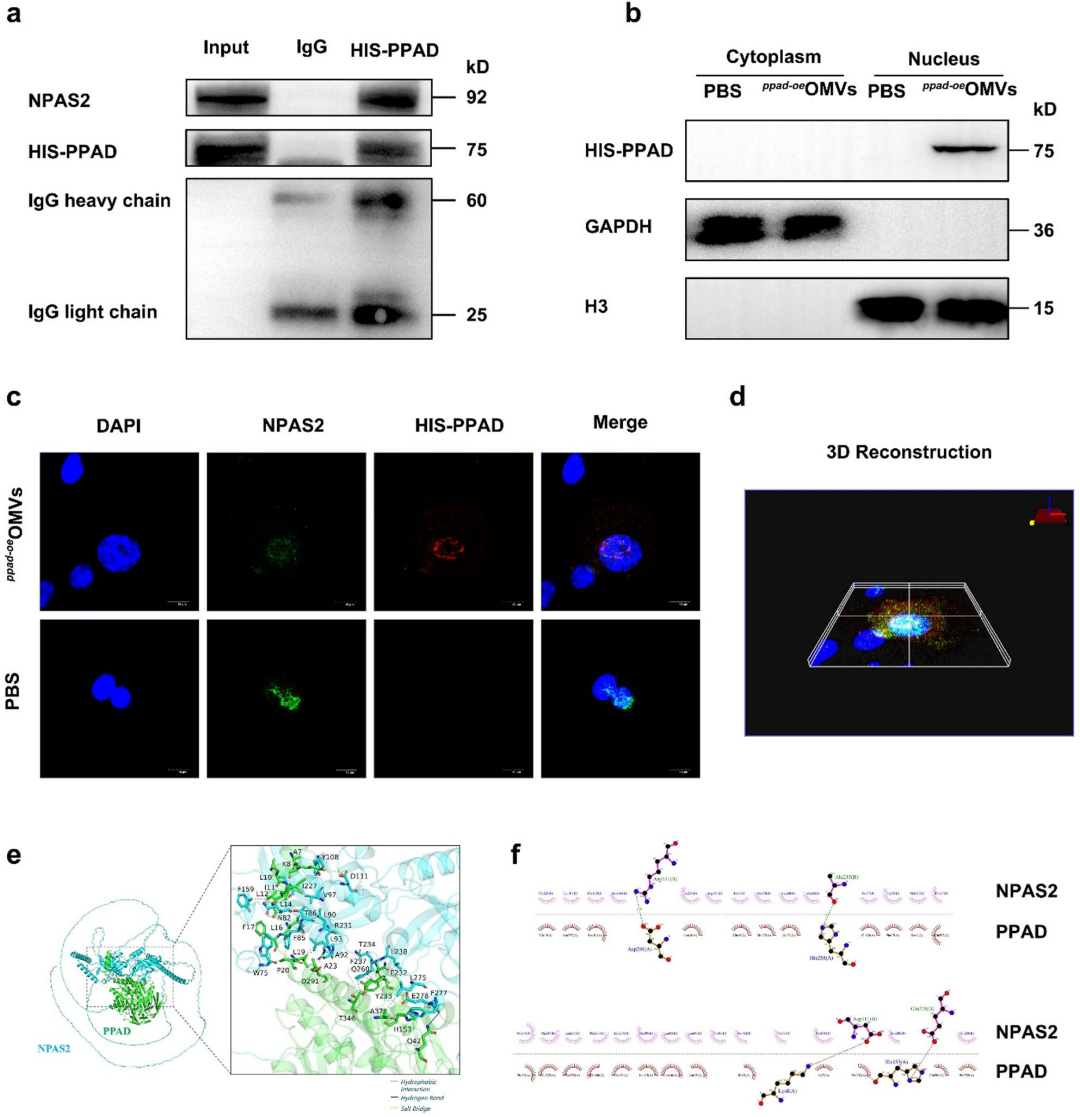
The binding mode of PPAD to NPAS2 may be through the binding of Asp291 of PPAD to Arg231 of Npas2. **a** Coimmunoprecipitation assay demonstrates the interaction between HIS-PPAD and NPAS2. **b** His-tagged-PPAD (HIS-PPAD) was detected in nucleus proteins of AML12 cells Treated with *P. gingivalis*^*ppad−oe*^ OMVs by western blot. **c**,** d** Immunofluorescence assay showed that NPAS2 and PPAD can co-locate in the nucleus. Scale bar = 10 μm. **e** Protein-protein docking of PPAD (green) and NPAS2 (blue). Gray dashed lines represent hydrophobic interactions, solid blue lines represent hydrogen bonds, yellow dashed lines represent salt Bridges, and yellow spheres represent valence electric centers. **f** Protein residue-residue 2D interaction diagram. The C atom is black, the O atom is red, the N atom is blue, the green dotted line represents the hydrogen bond, the green number represents the hydrogen bond length, the red dotted line represents the salt bridge, and the semi-circle is the amino acid residue that has hydrophobic interactions

To further explore the impact of PPAD on NPAS2 expression, we transfected *P. gingivalis* cells with a plasmid encoding His-tagged PPAD (HIS-PPAD) via electroporation, generating a PPAD-overexpressing strain (*P. gingivalis*^*ppad−oe*^), which was confirmed by agarose gel electrophoresis (Fig. S7). OMVs were isolated from the *P. gingivalis*^*ppad−oe*^ strain (Fig. S6b) and used to stimulate AML12 cells (^*ppad−oe*^OMV group). To biochemically validate the observed cellular colocalization, we performed a coimmunoprecipitation assay. In cells coexpressing His-tagged PPAD and NPAS2, immunoprecipitation of His-tagged PPAD resulted in the specific coprecipitation of NPAS2 (Fig. 9a), demonstrating the direct physical interaction between PPAD and NPAS2 in vivo. Western blot analysis of the cytoplasmic and nuclear proteins confirmed the presence of HIS-PPAD in the nuclear fractions (Fig. 9b). Notably, the clear detection of the HIS-PPAD band within the nuclear fractions not only supports the nuclear translocation of PPAD but also constitutes direct evidence of its successful high-level expression following transfection. The results of immunofluorescence assays further demonstrated the colocalization of NPAS2 and PPAD within the nucleus (Fig. 9c, d), suggesting that PPAD can enter the nucleus and bind to NPAS2. Binding energy analysis with PRODIGY revealed a strong interaction between PPAD and NPAS2, with a binding energy of -15.1 kcal/mol (Fig. 9e), indicating that the complex was stable. The mode of action, amino acid name and amino acid interaction site are shown in Appendix Table 6. The residue interaction diagram revealed the interaction of Asp291 of PPAD with Arg231 of NPAS2 as a potential binding mechanism (Fig. 9f).

## Discussion

Recent studies have revealed a significant positive correlation between periodontal disease and conditions associated with MASLD [6, 25]. MASLD and periodontitis have overlapping pathophysiological mechanisms and genetic signatures [26]. However, the causal link between periodontal disease and MASLD remains undetermined. A recent review revealed *P. gingivalis* infection as a direct risk factor for MASLD, a finding supported by clinical and experimental evidence. This bacterium disrupts metabolic, inflammatory, and immune homeostasis, causing systemic abnormalities [27]. Despite this association, the specific components of *P. gingivalis* that contribute to MASLD progression are unclear. This study introduces a novel concept: PPAD carried within *P. gingivalis* OMVs promotes lipid accumulation and exacerbates MASLD by inhibiting the NPAS2/CYP4A10 pathway.

Our clinical data establish a significant association between the severity of periodontitis and the presence of MASLD, suggesting a link at the level of integrated disease phenotypes. To extend this correlation and explore the potential driving mechanism underlying this association, we conducted complementary experimental investigations focusing on *P. gingivalis*, a key periodontal pathogen. The results of our in vivo experiments demonstrated that chronic *P. gingivalis* infection exacerbated hepatic steatosis, inflammation, and fibrosis in a MASLD mouse model. Corroborating these findings, the in vitro studies revealed that *P. gingivalis*-derived virulence factors (PPAD carried within *P. gingivalis* OMVs) can directly induce inflammatory and metabolic dysregulation in hepatocytes. Therefore, while our clinical analysis establishes a disease-level link, the experimental data advance the mechanistic understanding by identifying *P. gingivalis* as a plausible biological agent that can directly contribute to hepatic pathology, providing a causal relationship that connects oral dysbiosis to liver disease progression.

Various methods exist for establishing animal models to study periodontitis [28]. In this study, we used young C57BL/6J mice to establish the periodontitis model. Young mice are commonly selected for this application in the field, as they are in an active bone remodelling phase that can increase their susceptibility to experimentally induced inflammatory bone loss, thereby offering a sensitive system for evaluating the effects of *P. gingivalis* infection. Accordingly, we used oral implantation of *P. gingivalis* to establish our periodontitis model. In the *P. gingivalis* OMV infection model, we mimicked chronic, long-term periodontitis by maintaining the infection duration at eight weeks. Given the ability of *P. gingivalis* OMVs to enter the bloodstream and affect distant tissues, we used tail vein injections to establish the infection model, administering 5 µg of OMVs per injection three times weekly, as previously reported [19].

*P. gingivalis* may exacerbate MASLD progression through multiple mechanisms. First, dysbiotic oral pathogens can translocate to the gastrointestinal tract, causing gut dysbiosis. These pathogens and their toxic metabolites reach the liver via the portal vein, triggering adverse hepatic effects [29, 30]. Additionally, *P. gingivalis* can directly enter the bloodstream through ruptured periodontal pockets, eliciting a systemic inflammatory response [31]. This infection results in the release of pathogen-associated molecular patterns (PAMPs) and damage-associated molecular patterns (DAMPs) from periodontal tissues into the circulation. PAMPs, including peptidoglycan, lipopolysaccharides, gingipains, fimbriae, and bacterial DNA, along with DAMPs such as interleukin-1α, interleukin-1β, interleukin-8, and galectin-3, interact with hepatocytes and immune cells in the liver via pattern recognition receptors, specifically TLR-2 and TLR-4 [32]. In this study, we found that *P. gingivalis* accelerated MASLD progression and increased lipid accumulation in the livers of obese mice. However, in chow-fed mice, *P. gingivalis* primarily increased inflammation without significantly influencing hepatic fat accumulation. These results suggest that *P. gingivalis* amplifies lipid accumulation in livers already burdened with fat rather than inducing fat deposition in healthy livers, consistent with previous findings [13].

The results of transcriptome sequencing of mouse livers from the HFD and HFD-OMVs groups underscored the pivotal role of *Npas2* in metabolic regulation and circadian rhythm synchronization [33]. NPAS2 increases CYP1A2 expression by binding to the 416 bp E-box-like element in its promoter region, influencing its rhythmic activity in the liver [34]. Furthermore, CYP1A2 may disrupt lipid metabolism via the Pten/Akt/Srebp-1c signalling pathway [35], highlighting the importance of NPAS2 in maintaining lipoprotein homeostasis. Heatmap analysis also identified the altered expression of *Cyp4a10*, a homologue of human *CYP4A11* that catalyses the ω-hydroxylation of fatty acids in the liver and kidney, facilitating fatty acid metabolism [36]. On the basis of these findings, we hypothesized that *P. gingivalis* OMVs modulate hepatic lipid metabolism by inhibiting the NPAS2/CYP4A10 pathway, a hypothesis we tested in AML12 cells.

In this study, we found that PPAD carried within *P. gingivalis* OMVs promotes hepatic fat accumulation both in vitro and in vivo, potentially by inhibiting the NPAS2/CYP4A10 pathway. Our experimental evidence revealed that PPAD enters the nucleus and interacts with NPAS2. PPAD exists in two forms: a membrane-bound form and a soluble, secreted form [37, 38]. The soluble form is relatively rare, as PPAD is primarily released into the extracellular space within OMVs [39]. Structurally, full-length PPAD comprises four domains: an N-terminal signal peptide, a catalytic domain, an immunoglobulin superfamily domain (IgSF), and a C-terminal domain [40, 41]. Notably, the N-terminal and C-terminal regions of PPAD proteins carried within OMVs are truncated. These truncations confer a functional advantage by preventing autocitrullination, which is a vulnerability of the full-length variant [42].

Our NTA data, which are consistent with those in previous reports, indicate that PPAD deletion alters OMV biogenesis, resulting in the formation of smaller vesicles [43]. This observation raises an important consideration: the attenuated pathogenicity of *P. gingivalis*^*Δppad*^ OMVs observed in our models may not be attributable solely to the lack of PPAD enzymatic activity but could also be influenced by these biophysical alterations. Future studies are warranted to elucidate these contributions, for example, OMVs derived from a PPAD catalytic site mutant could be compared with those derived from the *P. gingivalis*^*Δppad*^ deletion strain, and by proteomic/lipidomic analyses could be performed to determine whether the OMV composition is also PPAD dependent.

PPAD-mediated citrullination has been implicated in systemic diseases such as rheumatoid arthritis, atherosclerosis, and Alzheimer’s disease [44]. Gingipain R cleaves host proteins to expose C-terminal arginines, which PPAD then citrullinates—establishing this activity as a critical mechanism underlying citrullination [22]. While earlier studies focused on inhibiting Gingipain R, recent findings suggest that targeting PPAD may be a more effective approach.

It is important to clarify that while the citrullination of host proteins by PPAD is a well-established mechanism in other pathological contexts, we did not directly quantify hepatic or systemic citrullination levels in our study. Our data demonstrated that infection with *P. gingivalis*, specifically the presence of its key virulence factor PPAD, was central to the exacerbation of MASLD phenotypes in our models. The established enzymatic function of PPAD provides a plausible and prioritized mechanistic hypothesis, potentially involving the modification of host proteins, for these observations. Future studies are needed to directly detect and quantify PPAD-mediated citrullination within the liver in the context of MASLD, which would be a critical step in validating this specific pathway.

Beyond the direct microbe–host interaction explored herein, the host immune response to periodontal pathogens, such as the generation of autoantibodies, including anti-citrullinated protein antibodies (ACPAs), constitutes another potential pathway linking periodontitis to systemic sequelae. Notably, ACPAs have been shown to directly impair hepatocyte function, promote hepatic inflammation, and activate profibrotic pathways [45, 46]. While ACPA levels were not measured in the present study, future investigations integrating serological analysis of both pathogen-specific antibodies and autoimmune markers such as ACPAs in MASLD cohorts could disentangle their respective contributions and potentially reveal a synergistic effect in driving disease progression.

### Limitations

This study has several limitations. First, this clinical cohort study was designed to assess the relationship between clinically diagnosed periodontitis and MASLD. Consequently, we did not measure serum antibody titres against specific periodontal pathogens, such as *P. gingivalis*. While this approach is valid for addressing the epidemiological association at the disease burden level, it precludes conclusions regarding the contribution of specific bacteria or the host’s humoral immune response to them. Future prospective studies that integrate clinical periodontal assessments with pathogen-specific serological assays and microbial analysis will be crucial for delineating the individual roles of key pathogenic members of the complex periodontal microbiome in MASLD pathogenesis.

While a standalone quantitative Western blot for PPAD was not performed, the overexpression and functional integrity of His-tagged PPAD were rigorously corroborated by its conspicuous detection in multiple subsequent assays: specifically, its visualization via immunofluorescence staining, the identification of its nuclear enrichment by nuclear fractionation, and the detection of its participation in specific protein‒protein interactions verified by coimmunoprecipitation. This multifaceted approach provides robust, albeit indirect, validation of successful transfection and protein expression.

## Conclusion

**Fig. 10:**
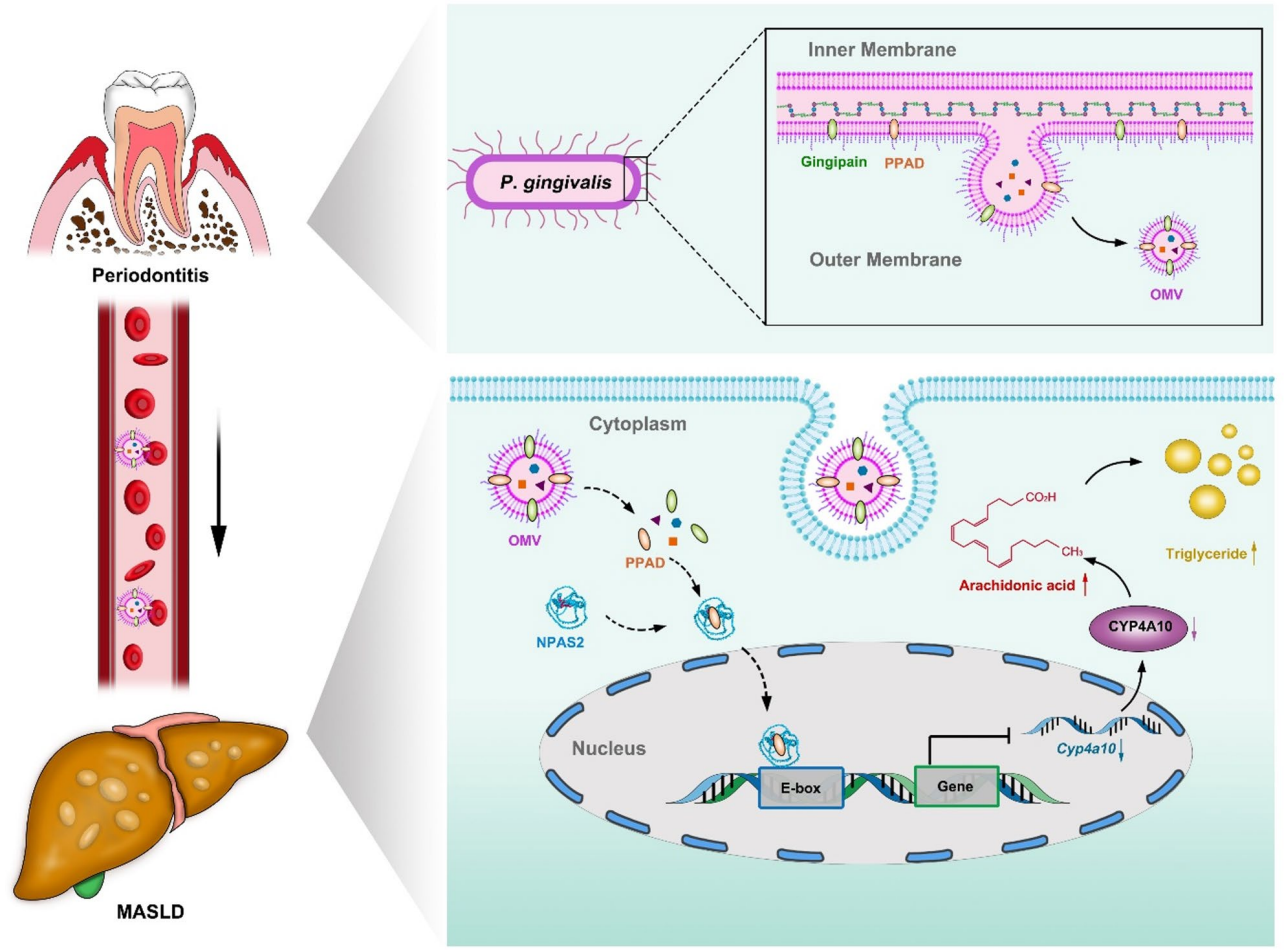
The hypothesis of the possible mechanism about the effect of *P. gingivalis* OMVs on MASLD Solid lines: experimentally supported; Dashed lines: hypothetical pathways

On the basis of our findings, we propose a working model (Fig. 10) in which PPAD carried within *P. gingivalis* OMVs may disrupt hepatic lipid metabolism via an interaction with NPAS2 and downregulation of CYP4A10. Specifically, we revealed a novel physical interaction between PPAD and the circadian regulator NPAS2. Although the precise functional consequence of this interaction requires further elucidation, it was consistently associated with significant downregulation of key metabolic genes in hepatocytes exposed to *P. gingivalis* OMVs. Future investigations are needed to determine whether PPAD directly modifies NPAS2—considering its noted enzymatic preference for C-terminal arginines—or alters its function through other mechanisms. Furthermore, gaining an understanding of how this interaction integrates with broader signalling networks to drive the observed transcriptional reprogramming and lipid accumulation is a crucial direction for subsequent research. Future investigations should aim to definitively establish whether the detrimental effects observed are mediated directly through the citrullination activity of PPAD on specific host targets. The confirmation of such a mechanism would provide a strong rationale for exploring PPAD inhibitor treatment as a novel therapeutic strategy aimed at disrupting the oral–systemic link in MASLD.

## Materials and methods

### Study population and sample collection

Participants in this study were recruited from the Stomatological Hospital of China Medical University, following approval by the institution’s Ethics Committee (K2024042). A total of 46 subjects were included in each of the two groups: the NO/Stage I–II periodontitis group and the Stage III–IV periodontitis group. The detailed inclusion and exclusion criteria are presented in Appendix Table 1.

The primary objective of the clinical component of this study was to investigate the association between periodontitis as a clinical disease entity and MASLD. Accordingly, the periodontal status was comprehensively assessed using standard clinical parameters, including the probing depth (PD), clinical attachment loss (CAL), bleeding on probing (BOP), and the plaque index (PI). This design was chosen to evaluate the holistic burden of periodontal inflammation in relation to MASLD severity. This study did not include microbiological assays or serological tests for specific periodontal pathogens, as the aim was to elucidate the association at the integrated clinical disease level rather than to attribute effects to a single pathogen. The participants were informed about the study’s purpose, risks, and benefits; completed a sociodemographic and medical questionnaire; and signed informed consent forms. The questionnaire collected data on demographic characteristics (e.g., age, sex, height, weight, and waist circumference), behavioural habits (smoking and alcohol consumption), and medical history (diabetes and hypertension). The clinical characteristics are presented in Appendix Table 2. During the initial visit, each participant underwent a comprehensive clinical examination, and a tailored treatment plan was created. Serum samples were collected for the assessment of triglycerides (TGs), total cholesterol (T-CHO), and liver function markers (ALT, AST, and gamma-glutamyltransferase (GGT)). The fatty liver index (FLI) and hepatic steatosis index (HSI), both of which are indicators of MASLD, were calculated [11]. Multivariate logistic regression was used to analyse the associations between periodontitis and the FLI/HSI after adjusting for age, sex, BMI, smoking status, alcohol consumption status, hypertension status, and diabetes status (Appendix Table 3).

### Animal experimental design

In the animal study, 8-week-old male wild-type C57BL/6J mice (SPF Biotechnology Co., Ltd, Beijing) were housed in a pathogen-free environment at the China Medical University Laboratory Animal Center and acclimatized for one week before the experiments. This protocol was approved by the Institutional Animal Care and Use Committee (CMU2023004).

For the animal experiments, a total of 24 mice were fed either a high-fat diet (HFD) or a standard chow diet (CD) (*n* = 12 mice per diet group). Within each diet group, the mice were subsequently randomly allocated to either the *P. gingivalis* infection or sham infection subgroup (final *n* = 6 mice per diet–treatment subgroup). For *P. gingivalis* treatment, *P. gingivalis* (10⁹ CFU/ml) suspended in 100 µl of PBS and 2% carboxymethyl cellulose was applied to the mandibular first molars three times weekly for 8 weeks to induce periodontitis [47]. The successful establishment of the periodontitis model is shown in Fig. S8. The experiment lasted for 20 weeks, with humane endpoints defined by signs of distress (e.g., panting, abnormal posture, or unkempt fur). Mice meeting these endpoint criteria were euthanized via CO₂ asphyxiation, which was also used at the time of tissue collection to minimize suffering.

A similar experimental setup was used to assess the effects of *P. gingivalis* outer membrane vesicles (OMVs) on hepatic lipid metabolism. For this study, 24 mice were divided into four groups: the CD, CD with OMVs (CD-OMVs), HFD, and HFD with OMVs (HFD-OMVs) groups. *P. gingivalis* OMVs (1 µg/µl, 5 µl per injection) were administered via the tail vein three times per week for 8 weeks to the mice in the OMV groups, while the mice in the control groups received PBS injections. Body weight was measured weekly, and the experiment was concluded at 20 weeks. Tissues were harvested following euthanasia via CO₂ asphyxiation.

### Bacterial culture and isolation of *p. gingivalis* omvs

The *P. gingivalis* W83 strain utilized in this study originated from the Oral Biology Department of the Stomatological Hospital of China Medical University. Bacterial cultures were maintained under anaerobic conditions (37 °C) using brain heart infusion (BHI) agar supplemented with 5% sterilized defibrinated sheep blood, 5 µl/ml hemin solution, and 1 µl/ml vitamin K solution.

The *P. gingivalis*^*Δppad*^ mutant, generated via allelic exchange as previously described [48], was obtained from Associate Professor Liu Jingbo. The genotype was verified in house by PCR and sequencing.

To generate the overexpression strain, the ppad gene was cloned and inserted into the pET-22b(+) vector to create a C-terminal His tag fusion (pET-22b(+)-PPAD). This plasmid was subsequently electroporated into the *P. gingivalis*^*Δppad*^ mutant. The expression of the recombinant His-PPAD protein was confirmed by Western blot analysis with an anti-His tag antibody.

Outer membrane vesicles (OMVs) were isolated with an ExoBacteria OMV Isolation Kit (System Biosciences LLC, CA 94303), a commercial system optimized for gram-negative species. The purified OMV pellet was resuspended in sterile PBS. To determine the protein concentration, OMVs were lysed with 1% SDS, and the total protein content was measured using a bicinchoninic acid (BCA) assay kit (Thermo Fisher Scientific, USA) according to the manufacturer’s instructions. The OMV stock concentration was calculated and expressed as µg of total protein per ml (µg/µl). All subsequent in vitro and in vivo treatments were performed with OMV concentrations normalized to this protein concentration. After isolation, the OMV suspensions were normalized to a concentration of 1 µg/µl with PBS and cryopreserved at -80 °C until experimental use. Structural characterization of OMVs was conducted by transmission electron microscopy (TEM; Thermo Talos L120C G2, USA), while nanoparticle tracking analysis (NTA; ZetaView PMX-120, Particle Metrix, Germany) was used to determine the vesicle size distribution in the liquid phase.

The morphological characteristics of the isolated exosomes were analysed by TEM (Thermo Talos L120C G2, USA). Additionally, the sizes of the exosome particles in suspension were measured using NTA (Particle Metrix ZetaView, Germany).

### Labelling and tracking of *p. gingivalis* omvs

The isolated OMVs were labelled by incubation with 1% (v/v) DiD (Beyotime Biotechnology, China) at 37 °C for 30 min. Excess DiD not bound to *P. gingivalis* OMVs was removed using Exosome Spin Columns (Liaoning Rengen Biosciences Co., Ltd). PBS treated with the dye under identical conditions served as a negative control.

For in vivo studies, C57BL/6J male mice (8 weeks old) were injected via the tail vein with 15 µg of DiD-labelled OMVs. Then, 2, 6, 12, and 24 h post-injection, mice were sacrificed, and organ-specific (liver, heart, lung, kidney, and spleen) DiD fluorescence was analysed using an IVIS Spectrum imaging system (Caliper Life Sciences).

After the mice were placed under deep anaesthesia, transcardial perfusion was performed with prechilled PBS followed by 4% paraformaldehyde. The livers were extracted, fixed with paraformaldehyde at 4 °C, and processed for paraffin embedding. The tissue sections were blocked and incubated with an anti-HNF-4α antibody (Abcam, Cambridge, UK) and an AlexaFluor 488-conjugated goat anti-rabbit secondary antibody (Abcam, Cambridge, UK) prior to DAPI staining. Imaging was conducted using a C2 confocal laser scanning microscope (Nikon, Japan).

For in vitro studies, AML12 cells were treated with 2 µg of DiD-labelled OMVs for 6 h, fixed with 4% paraformaldehyde, and stained with phalloidin for visualization of the cytoskeleton. Coverslips were mounted using ProLong Gold reagent with DAPI, and the cells were analysed using a Nikon C2 confocal microscope.

### Histological analyses

Mouse livers were fixed with 4% paraformaldehyde, embedded in paraffin, sectioned at a thickness of 5 μm, and stained with haematoxylin and eosin for histological scoring. A three-stage scoring system was used to assess steatosis (0–3), lobular inflammation (0–3), and hepatocyte ballooning (0–2) [49]. Fibrosis was quantified by measuring the area of collagen deposition on Masson’s trichrome-stained sections. For each sample, five random high-power fields (200× magnification) were imaged. The blue-stained collagen-containing area was then quantified as a percentage of the total tissue area using image analysis software (ImageJ, National Institutes of Health, Bethesda, MD, USA). The average percentage over the five fields was used as the fibrosis index for each mouse. Additionally, frozen liver samples (-20 °C) were sliced into 10 μm sections and stained with Oil Red O to assess lipid accumulation. Images were acquired using a Pannoramic Slide Scanner (3D HISTECH, Hungary).

### Biochemical analyses of serum and hepatic factors

The TG and T-CHO contents in liver tissues and the serum concentrations of T-CHO, TG, ALT, AST and GGT were measured using assay kits (Jiancheng Bioengineering Institute, China) according to the manufacturer’s protocols.

### Quantitative reverse transcription—polymerase chain reaction

Cellular RNA extraction was performed using TRIzol reagent (Invitrogen, MD, USA) following standard protocols. Quantitative reverse transcription–PCR (qRT‒PCR) analyses were performed in an ABI Prism 7500 system (Applied Biosystems, CA, USA) with SYBR Premix Ex Taq™ II (Takara Bio, China). The primer sequences are detailed in Appendix Table 5, and GAPDH was used as the endogenous control for signal normalization. Relative mRNA expression was quantified by the comparative threshold cycle (2 − ΔΔCT) method.

### Western blotting

Western blotting was performed using a Bio–Rad protein analysis system (Bio–Rad, USA). Primary antibodies against SREBP1, PPARγ, CYP4A, and NPAS2 (all from Abcam, Cambridge, UK) and GAPDH (Proteintech, Wuhan, China) were used. Fluorescent secondary antibodies (Proteintech, Wuhan, China) were used for detection, and an Odyssey CLx infrared imaging system (LI-COR, USA) was used to visualize the protein bands. Semiquantitative analysis was conducted with ImageJ v1.52 software (NIH, Bethesda, MD, USA).

### Cell culture and treatments

AML12 cells (Pricella Biotechnology Co., Ltd, Wuhan, China) were cultured in DMEM supplemented with 10% FBS and ITS-G (5 mg/ml insulin, 5 mg/L transferrin, and 5 µg/L selenous acid; Pricella Biotechnology, Wuhan, China) at 37 °C in 5% CO₂. To prepare the PO solution, oleic acid and palmitic acid were dissolved in 75% ethanol at 55 °C; diluted in DMEM containing 1% fatty acid-free BSA (Sigma‒Aldrich, A8806) to final concentrations of 500 µM and 250 µM, respectively; and sterilized using 0.2 μm filters. An in vitro MASLD model was established by treating AML12 cells with the solution containing palmitic acid (250 µM) and oleic acid (500 µM) for 24 h, as described in established protocols, to induce lipid accumulation and cellular lipotoxicity [50].

To generate AML12 cells stably overexpressing NPAS2, the full-length mouse Npas2 cDNA was cloned and inserted into the GV657 vector (GeneChem), resulting in CMV promoter-driven expression of an NPAS2 protein with a C-terminal fusion of the 3×Flag tag. The same vector encoded the ZsGreen fluorescent protein and a puromycin resistance gene under the control of a separate EF1α promoter. AML12 cells were transfected with the recombinant plasmid or the empty vector control using Lipofectamine 3000 reagent (Thermo Fisher Scientific). To establish a stable polyclonal population, transfected cells were selected with 2 µg/ml puromycin for 14 days, during which the medium was replaced every 3 days.

### Nile red staining

Nile red staining of AML12 hepatocytes was performed as described previously [51]. Briefly, the cells were fixed with 10% formaldehyde, stained with 1 µg/ml Nile red at 4 °C for 30 min, and then washed with PBS. Fluorescence was visualized using microscopy, and flow cytometry was performed for quantification (excitation: 530 nm; emission: 590 nm).

### Protein–protein docking

The protein structures of PPAD (UniProt ID: A0AAE9XC92) and NPAS2 (UniProt ID: P97460) were retrieved from the UniProt database. AlphaFold 3 was used for protein modelling on the basis of the full-length sequences. The binding energy of the PPAD–NPAS2 complex was calculated with PRODIGY, and LigPlot was used to generate a 2D interaction map. PyMOL was used for 3D visualization of the binding interface for systematic analysis.

### Coimmunoprecipitation (Co-IP) assay

Protein‒protein interactions were analysed using a commercial coimmunoprecipitation kit (Absin, Shanghai, China). AML12 cells treated with PBS or *P. gingivalis*^*ppad-oe*^ lysate were lysed. Proteins recovered from the lysates were incubated overnight at 4 °C with anti-His tag antibody-conjugated magnetic beads. After being washed, the bound proteins were eluted and analysed by Western blotting to detect coprecipitated NPAS2, with IgG used as the negative control.

### RNA-seq analysis

Liver transcriptome analysis was performed on mice from the HFD-OMVs and HFD control groups (*n* = 3 mice per group). Total RNA was extracted from liver tissues using TRIzol reagent. We performed 2 × 150 bp paired-end sequencing (PE150 mode) on the Illumina NovaSeq™ 6000 platform (LC-Bio Technology Co., Ltd, Hangzhou, China) following the vendor’s recommended protocol. The specific steps were as follows: Following quality control (RIN > 7.0, NanoDrop A260/A280 > 1.8), poly(A) mRNA was enriched, fragmented, and used for library construction. Sequencing was performed on the Illumina NovaSeq 6000 platform (PE150 mode, > 30 M reads/sample). Clean reads were aligned to the mouse genome (GRCm39) using HISAT2, and transcript quantification was performed with StringTie. Differential expression analysis was conducted with DESeq2, applying thresholds of |log2FC| > 1 and adjusted *p* < 0.05. Gene set enrichment analysis (GSEA) was performed to identify significantly enriched pathways.

### Immunofluorescence and costaining

To analyse the colocalization of PPAD and NPAS2, immunofluorescence staining was performed. Cultured cells were fixed, permeabilized, and blocked. The cells were then incubated overnight at 4 °C with the following primary antibodies: a mouse monoclonal anti-His tag antibody (AE003, ABclonal) to detect the overexpressed His-tagged PPAD protein and a rabbit monoclonal anti-NPAS2 antibody (A16930, ABclonal). After being washed, the cells were incubated with the corresponding fluorescent secondary antibodies: Alexa Fluor 594-conjugated goat anti-moused IgG (RGAM004; Proteintech) and Alexa Fluor 488-conjugated goat anti-rabbit IgG (RGAR002; Proteintech). The cell nuclei were counterstained with DAPI. Fluorescence images were acquired using a confocal laser scanning microscope.

### Statistical analysis

All the quantitative data are presented as the mean ± standard deviation (SD) values. The normality of the data distribution was assessed using the Shapiro‒Wilk test, and the homogeneity of variance was evaluated with Levene’s test. On the basis of these assessments, the appropriate parametric or nonparametric statistical tests were selected for group comparisons. For comparisons between two independent groups of data meeting the assumptions of normality and equal variance, unpaired two-tailed Student’s t test was applied. For comparisons among three or more independent groups of data meeting the same assumptions, one-way analysis of variance (ANOVA) was performed. Whenever a significant overall difference was indicated by ANOVA (p < 0.05), Tukey’s honest significant difference (HSD) post hoc test was conducted for all pairwise comparisons. Data that deviated significantly from a normal distribution or were ordinal in nature were analysed using nonparametric tests: the Mann‒Whitney U test for two-group comparisons or the Kruskal‒Wallis test followed by Dunn’s post hoc test for multiple-group comparisons. The threshold for statistical significance was set at p < 0.05. All analyses were conducted using GraphPad Prism 9.0 (GraphPad Software, Inc., San Diego, CA, USA).

## Electronic Supplementary Material

Below is the link to the electronic supplementary material.

Supplementary Material 1.

Supplementary Material 2.

## Data Availability

The data associated with this paper are available upon request to the corresponding author.
