# Supplementary material for: Peptidylarginine deiminase in Porphyromonas gingivalis-derived outer membrane vesicles exacerbates metabolic dysfunction-associated steatotic liver disease through the NPAS2/CYP4A10 pathway
Source: J Nanobiotechnology. 2026 May 9;24:614. doi: 10.1186/s12951-026-04523-x (PMC13330145; doi:10.1186/s12951-026-04523-x)
Supplement: Supplementary file 1 — Supplementary Material 1. [file 12951_2026_4523_MOESM1_ESM.docx]

**Appendix**

**Appendix Table 1 Inclusion and exclusion criteria**

| **Inclusion criteria** |
| --- |
| NO/Stages I–II periodontitis group:   1. Han population aged 20 years or older. 2. Maximum CAL on proximal surfaces ≤ 4 mm, PPD≤ 5 mm, no tooth loss due to periodontitis, absence of grade II or III furcation involvement, and RBL not exceeding 1/3 of the length of the root.   Stage III–IV periodontitis group:   1. Han population aged 20 years or older. 2. Maximum CAL on proximal surfaces > 5 mm, PPD > 6 mm, presence of tooth loss due to periodontitis, grade II or III furcation involvement, and RBL exceeding 1/3 of the length of the root. |
| **Exclusion criteria** |
| 1. Received periodontal treatment in the past 1 year. 2. Received antibiotic treatment in the past 6 months. 3. Received non-steroidal anti-inflammatory drugs within 3 months. 4. Suffer from uncontrolled severe systemic diseases. 5. Suffer from other serious oral diseases. 6. Heavy smoker (> 10 PCS/day). 7. Pregnant or nursing. 8. People with a special diet preference (vegan or pure meat eaters). |

Abbreviations: PPD, periodontal probing depth; CAL, clinical attachment loss; RBL, radiographic bone loss.

**Appendix Table 2 Clinical characteristics of the participants according to their periodontal diagnosis.**

| Characteristics | Periodontal status | | *P* value |
| --- | --- | --- | --- |
|  | NO/Stages I–II Periodontitis | Stages III–IV Periodontitis |  |
| Age (years) | 30.50 (25.75, 35.25) | 48.00 (38.50, 58.25) | <0.001 |
| Sex, n (%) |  |  | 0.037 |
| female | 18 (39.13) | 28 (60.87) |  |
| male | 28 (60.87) | 18 (39.13) |  |
| BMI (kg/m^2^) | 23.12 ± 2.73 | 24.27 ± 2.67 | 0.045 |
| Smoking status, n (%) |  |  | 0.01 |
| Never smoker | 43 (93.48) | 32 (69.57) |  |
| Former smoker | 0 (0) | 3 (6.52) |  |
| Current smoker | 3 (6.52) | 11 (23.91) |  |
| Drinking status, n (%) |  |  | <0.001 |
| Non-drinker | 46 (100) | 29 (63.04) |  |
| Light drinker | 0 (0) | 12 (26.09) |  |
| Moderate drinker | 0 (0) | 5 (10.87) |  |
| Hypertension status, n (%) |  |  | 0.024 |
| No | 44 (95.65) | 37 (80.43) |  |
| Yes | 2 (4.35) | 9 (19.57) |  |
| Diabetes status, n (%) |  |  | 0.153 |
| No | 46 (100) | 44 (95.65) |  |
| Yes | 0 (0) | 2 (4.35) |  |
| Missing teeth, n | 0.00 (0.00, 0.00) | 1.00 (0.00, 3.00) | < 0.001 |
| PPD, mm | 2.13 (1.91, 2.35) | 3.92 (3.29, 4.61) | < 0.001 |
| CAL, mm | 2.17 (1.96, 2.42) | 4.13 (3.51, 4.83) | < 0.001 |
| BOP (%) | 14.59 (4.19, 23.21) | 80.66 (49.67, 97.77) | < 0.001 |
| TG, mmol/L | 0.62 (0.40, 1.36) | 1.13 (0.68, 1.95) | < 0.001 |
| T-CHO, mmol/L | 4.17 ± 0.73 | 4.48 ± 0.99 | 0.089 |
| HSI, continuous | 39.82 (33.66, 47.93) | 40.84 (33.99, 49.12) | 0.882 |
| HSI, n (%) |  |  | 0.822 |
| ≥36 | 32 (69.57) | 31 (67.39) |  |
| <36 | 14 (30.43) | 15 (32.61) |  |
| FLI, continuous | 26.14 (14.60, 44.39) | 64.72 (36.68, 82.69) | < 0.001 |
| FLI, n (%) |  |  | < 0.001 |
| ≥60 | 8 (17.39) | 26 (56.52) |  |
| <60 | 38 (82.61) | 20 (43.48) |  |

Abbreviations: BMI, body mass index; PPD, periodontal probing depth; CAL, clinical attachment loss; BOP, bleeding on probing; TG, triglyceride; T-CHO, total cholesterol; HSI, hepatic steatosis index; FLI, fatty liver index.

**Appendix Table 3** U**nivariate analysis of HSI and FLI.**

| **Characteristics** | | **HSI** | ***P* value** |
| --- | --- | --- | --- |
| Age (years) | | 0.992 (0.961, 1.203) | 0.607 |
| Sex, n (%) | |  | 0.037 |
| male | | ref | ref |
| female | | 0.904 (0.375, 2.180) | 0.822 |
| BMI (kg/m^2^) | | 1.088 (0.923, 1.282) | 0.314 |
| Smoking status, n (%) | |  |  |
| Never smoker | | ref | ref |
| Former smoker | | - | 0.999 |
| Current smoker | | 1.622 (0.413, 6.368) | 0.488 |
| Drinking status, n (%) | |  |  |
| Non-drinker | | ref | ref |
| Light drinker | | 2.353 (0.478, 11.582) | 0.293 |
| Moderate drinker | | 0.314 (0.049, 2.003) | 0.220 |
| Hypertension status, n (%) | |  |  |
| No | | ref | ref |
| Yes | | 0.505 (0.141, 1.816) | 0.296 |
| Diabetes status, n (%) | |  |  |
| No | | ref | ref |
| Yes | | - | 0.999 |
| Missing teeth, n | | 0.988 (0.813, 1.201) | 0.902 |
| PPD, mm | | 1.118 (0.780, 1.601) | 0.545 |
| CAL, mm | | 1.005 (0.765, 1.321) | 0.971 |
| BOP (%) | | 0.997 (0.984, 1.009) | 0.594 |
| TG, mmol/L | | 0.866 (0.554, 1.353) | 0.526 |
| T-CHO, mmol/L | | 0.810 (0489, 1.341) | 0.810 |
| Periodontitis | |  |  |
| NO/Stages I–II Periodontitis | | ref | ref |
| Stages III–IV Periodontitis | | 0.904 (0.375, 2.180) | 0.822 |
| **Characteristics** | | **FLI** | ***P* value** |
| Age (years) | | 1.065 (1.027, 1.104) | 0.001 |
| Sex, n (%) | |  |  |
| male | ref | ref |  |
| female | 0.255 (0.103, 0.632) | 0.003 |  |
| BMI (kg/m^2^) | 1.461 (1.198, 1.783) | <0.001 |  |
| Smoking status, n (%) |  |  |  |
| Never smoker | ref | ref |  |
| Former smoker | - | 0.999 |  |
| Current smoker | 6.429 (1.816, 22.762) | <0.001 |  |
| Drinking status, n (%) |  |  |  |
| Non-drinker | ref | ref |  |
| Light drinker | 2.125 (0.620, 7.279) | 0.230 |  |
| Moderate drinker | 8.500 (0.901, 80.193) | 0.062 |  |
| Hypertension status, n (%) |  |  |  |
| No | ref | ref |  |
| Yes | 3.500 (0.942, 13.004) | 0.061 |  |
| Diabetes status, n (%) |  |  |  |
| No | ref | ref |  |
| Yes | - | 0.999 |  |
| Missing teeth, n | 1.188 (0.974, 1.448) | 0.089 |  |
| PPD, mm | 1.518 (1.054, 2.187) | 0.025 |  |
| CAL, mm | 1.469 (1.085, 1.987) | 0.013 |  |
| BOP (%) | 1.019 (1.006, 1.032) | 0.005 |  |
| TG, mmol/L | 16.370 (5.218, 51.353) | < 0.001 |  |
| T-CHO, mmol/L | 0.922 (0.567, 1.498) | 0.742 |  |
| Periodontitis |  |  |  |
| NO/Stages I–II Periodontitis | ref | ref |  |
| Stages III–IV Periodontitis | 6.175 (2.365, 16.123) | < 0.001 |  |

Abbreviations: BMI, body mass index; PPD, periodontal probing depth; CAL, clinical attachment loss; BOP, bleeding on probing; TG, triglyceride; T-CHO, total cholesterol; HSI, hepatic steatosis index; FLI, fatty liver index.

**Appendix Table 4** **HR (95% CI) for FLI or HSI according to PPD, CAL, BOP.**

| Outcome | MASLD | | *P* value |
| --- | --- | --- | --- |
|  | NO (ref) | Yes |  |
| **HSI > 36** |  |  |  |
| PPD |  |  |  |
| Model 0^a^ | 1.00 | 1.118 (0.780, 1.601) | 0.545 |
| Model 1^b^ | 1.00 | 1.185 (0.789, 1.779) | 0.413 |
| Model 2^c^ | 1.00 | 1.368 (0.811, 2.308) | 0.240 |
| Model 3^d^ | 1.00 | 1.314 (0.774, 2.230) | 0.312 |
| CAL |  |  |  |
| Model 0 ^a^ | 1.00 | 1.005 (0.765, 1.321) | 0.971 |
| Model 1 ^b^ | 1.00 | 1.028 (0.756, 1.397) | 0.861 |
| Model 2 ^c^ | 1.00 | 1.135 (0.785, 1.642) | 0.500 |
| Model 3^d^ | 1.00 | 1.098 (0.758, 1.591) | 0.622 |
| BOP |  |  |  |
| Model 0 ^a^ | 1.00 | 0.997 (0.984, 1.009) | 0.594 |
| Model 1 ^b^ | 1.00 | 0.997 (0.982, 1.012) | 0.661 |
| Model 2 ^c^ | 1.00 | 0.998 (0.980, 1.015) | 0.795 |
| Model 3^d^ | 1.00 | 0.996 (0.978, 1.014) | 0.341 |
| **FLI > 60** |  |  |  |
| PPD |  |  |  |
| Model 0 ^a^ | 1.00 | 1.518 (1.054, 2.187) | 0.025 |
| Model 1 ^b^ | 1.00 | 1.070 (0.671, 1.706) | 0.777 |
| Model 2 ^c^ | 1.00 | 0.990 (0.578, 1.698) | 0.972 |
| Model 3^d^ | 1.00 | 0.989 (0.565, 1.734) | 0.971 |
| CAL |  |  |  |
| Model 0 ^a^ | 1.00 | 1.469 (1.085, 1.987) | 0.013 |
| Model 1 ^b^ | 1.00 | 1.049 (0.712, 1.546) | 0.809 |
| Model 2 ^c^ | 1.00 | 0.965 (0.609, 1.527) | 0.878 |
| Model 3^d^ | 1.00 | 0.951 (0.589, 1.537) | 0.839 |
| BOP |  |  |  |
| Model 0 ^a^ | 1.00 | 1.019 (1.006, 1.032) | 0.005 |
| Model 1 ^b^ | 1.00 | 1.006 (0.987, 1.024) | 0.552 |
| Model 2 ^c^ | 1.00 | 1.003 (0.982, 1.024) | 0.782 |
| Model 3^d^ | 1.00 | 1.002 (0.981, 1.024) | 0.839 |

Abbreviations: HR, hazard ratio; CI, confidence interval; MASLD, metabolic dysfunction-associated steatotic liver disease; HSI, hepatic steatosis index; FLI, fatty liver index; PPD, periodontal probing depth; CAL, clinical attachment loss; BOP, bleeding on probing.

^a^ Model 0 was univariate analysis.

^b^ Model 1 included age, sex (male or female), and BMI.

^c^ Model 2, based on Model 1, we further adjusted for smoking status (never smokers, former, or current smokers), drinking status (non-drinker, light drinker, moderate drinker).

^d^ Model 3, based on Model 2, we further adjusted for systemic diseases associated with periodontitis including hypertension status (no, or yes) and diabetes status (no, or yes).

**Appendix Table 5 Primers for quantitative PCR**

| **Gene name** | **Forward primer sequence (5’-3’)** | **Reverse primer sequence (5’-3’)** |
| --- | --- | --- |
| *Acadl* | CTTCAGCCTCCACTCAGATATTGTC | TACACTTGCCCGCCGTCATC |
| *Acca* | TATGGAAGTCGGCTATGGAAATTGC | TTGTCAGGAAGAGGCGGATGG |
| *Acox1* | GGTGGTATGGTGTCGTACTTGAATG | TGTAGGCTTCTGTCAGGCTGTC |
| *Cpt1a* | CACAACAACGGCAGAGCAGAG | ACACCACATAGAGGCAGAAGAGG |
| *Cyp2a4* | CGGAGAAGGACTGGCTAGGATG | TGTAGGTTGGTGGGATCGTGAC |
| *Cyp4a10* | GCACCAGATTCTTCTCACCATAGC | TCAAAGCGGAGCAGGGTCAG |
| *Elovl6* | ATCTGATGAACAAGCGAGCCAAG | AAGAGCACCGAATATACTGAAGACG |
| *Fasn* | TCCTGAAGCCGAACACCTCTG | GCGACAATATCCACTCCCTGAATC |
| *Gapdh* | AGGTCGGTGTGAACGGATTTG | TGTAGACCATGTAGTTGAGGTCA |
| *Gapdh* | AGGTCGGTGTGAACGGATTTG | TGTAGACCATGTAGTTGAGGTCA |
| *Itgam* | GCATCAATAGCCAGCCTCAGTG | AGCCAGGTCCATCAAGCCATC |
| *Npas2* | CACTACTACATCACCTACCACCAAT | CATCTGCGTAACTGACCACTGAG |
| *Ppara* | CGGGAAAGACCAGCAACAACC | AGCAGTGGAAGAATCGGACCTC |
| *Pparg* | TGTTCGCCAAGGTGCTCCAG | GCTCATGTCTGTCTCTGTCTTCTTG |
| *Ppat* | AGGCGGACTTGGTTAGCACAG | CGGTTCTTACACAGCACTTCCAC |
| *Scd1* | CAGCCTGTTCGTTAGCACCTTC | GGGATTGAATGTTCTTGTCGTAGGG |
| *Serpine1* | AGAATCCCACACAGCCCATCAG | CATAGCCAGCACCGAGGACAC |
| *Srebp1* | GGATCGCAGTCTGAGGAGGAG | CCAGGAGCCGACAGGAAGG |

**Appendix Table 6 Interaction mode, amino acid name and location of PPAD and NPAS2 protein**

| **Hydrophobic Interactions** | | |  |  |  |  |
| --- | --- | --- | --- | --- | --- | --- |
| RESNR | RESTYPE | RESCHAIN | RESNR | RESTYPE | RESCHAIN | DIST(Å) |
| 7 | ALA | PPAD | 108 | TYR | NPAS2 | 3.28 |
| 8 | LYS | PPAD | 108 | TYR | NPAS2 | 3.73 |
| 10 | LEU | PPAD | 227 | ILE | NPAS2 | 2.9 |
| 11 | ILE | PPAD | 108 | TYR | NPAS2 | 2.56 |
| 11 | ILE | PPAD | 97 | VAL | NPAS2 | 3.32 |
| 11 | ILE | PPAD | 97 | VAL | NPAS2 | 3.4 |
| 12 | LEU | PPAD | 82 | ASN | NPAS2 | 3.96 |
| 14 | LEU | PPAD | 159 | PHE | NPAS2 | 3.55 |
| 14 | LEU | PPAD | 227 | ILE | NPAS2 | 3.08 |
| 16 | LEU | PPAD | 86 | THR | NPAS2 | 3.08 |
| 16 | LEU | PPAD | 90 | LEU | NPAS2 | 3.77 |
| 17 | PHE | PPAD | 75 | TRP | NPAS2 | 3.64 |
| 17 | PHE | PPAD | 75 | TRP | NPAS2 | 2.65 |
| 19 | LEU | PPAD | 93 | LEU | NPAS2 | 3.88 |
| 19 | LEU | PPAD | 93 | LEU | NPAS2 | 2.64 |
| 20 | PRO | PPAD | 85 | PHE | NPAS2 | 3.22 |
| 20 | PRO | PPAD | 75 | TRP | NPAS2 | 2.69 |
| 23 | ALA | PPAD | 92 | ALA | NPAS2 | 3.88 |
| 37 | ALA | PPAD | 275 | LEU | NPAS2 | 3.59 |
| 232 | GLU | PPAD | 237 | PHE | NPAS2 | 3.22 |
| 232 | GLU | PPAD | 237 | PHE | NPAS2 | 3.05 |
| 233 | TYR | PPAD | 237 | PHE | NPAS2 | 3.46 |
| 233 | TYR | PPAD | 237 | PHE | NPAS2 | 2.48 |
| 233 | TYR | PPAD | 238 | LEU | NPAS2 | 3.76 |
| 260 | GLN | PPAD | 234 | THR | NPAS2 | 3.92 |
| 346 | THR | PPAD | 237 | PHE | NPAS2 | 3.1 |
| **Hydrogen Bonds** | |  |  |  |  |  |
| RESNR | RESTYPE | RESCHAIN | RESNR | RESTYPE | RESCHAIN | DIST(Å) |
| 42 | GLN | PPAD | 277 | PHE | NPAS2 | 3.02 |
| **Salt Bridges** | |  |  |  |  |  |
| RESNR | RESTYPE | RESCHAIN | RESNR | RESTYPE | RESCHAIN | DIST(Å) |
| 8 | LYS | PPAD | 111 | ASP | NPAS2 | 4.33 |
| 153 | HIS | PPAD | 278 | GLU | NPAS2 | 4.03 |
| 291 | ASP | PPAD | 231 | ARG | NPAS2 | 3.98 |
