# Supplementary material for: Peptidylarginine deiminase in Porphyromonas gingivalis-derived outer membrane vesicles exacerbates metabolic dysfunction-associated steatotic liver disease through the NPAS2/CYP4A10 pathway
Source: J Nanobiotechnology. 2026 May 9;24:614. doi: 10.1186/s12951-026-04523-x (PMC13330145; doi:10.1186/s12951-026-04523-x)
Supplement: Supplementary file 2 — Supplementary Material 2. [file 12951_2026_4523_MOESM2_ESM.pdf]

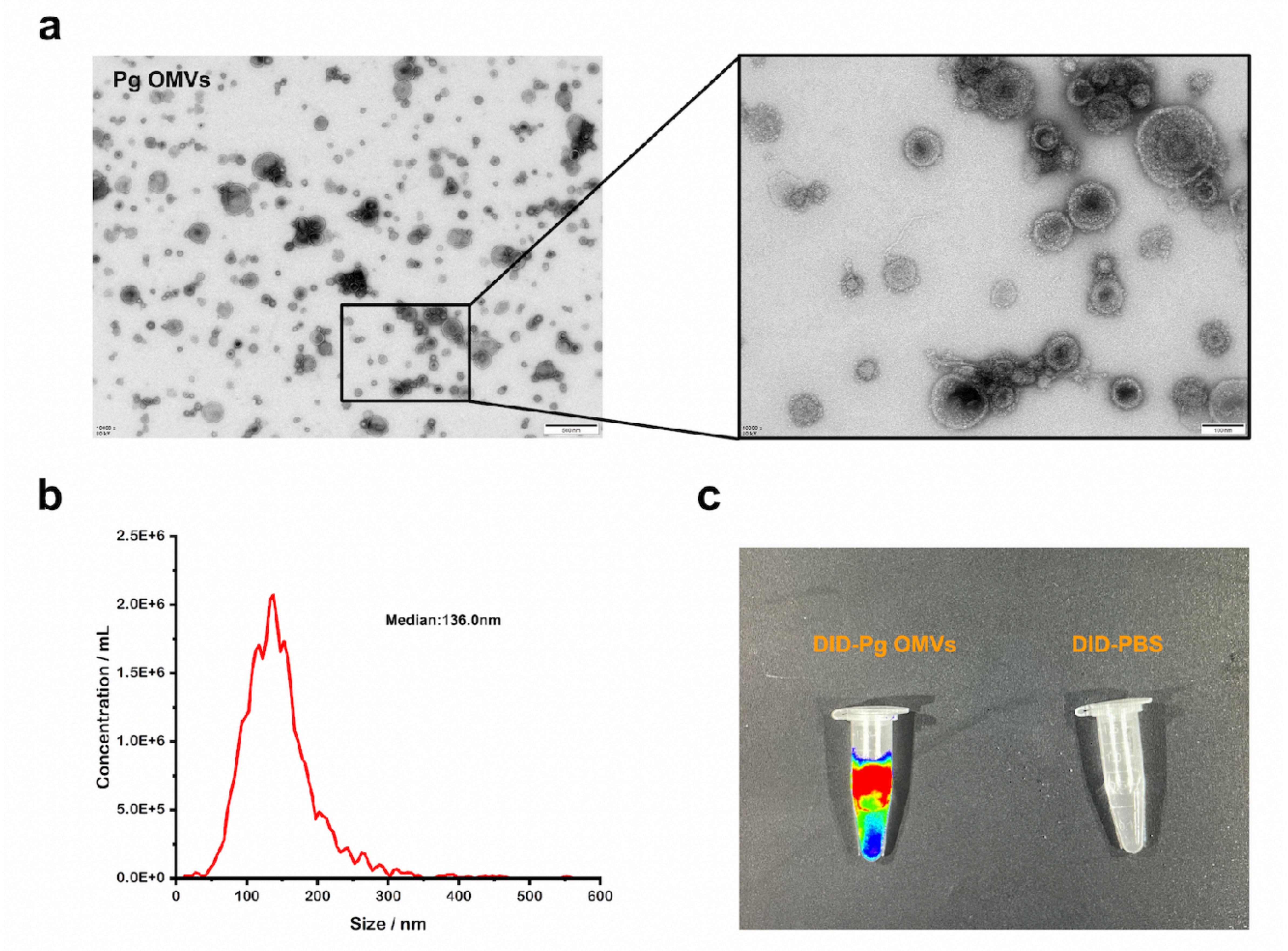

Fig. S1 Extraction, identification and labeling of *P. gingivalis* OMVs.

a Transmission electron micrographs of the structure of *P. gingivalis* OMVs. Scale bar = 500 nm, Scale bar = 100 nm. **b** Particle size of *P. gingivalis* OMVs measured by NTA. **c** The image of DiD-labeled *P. gingivalis* OMVs and DiD-labeled PBS by IVIS Spectrum imaging system. OMVs, outer membrane vesicles; NTA, Nanoparticle tracking analysis.

a

b

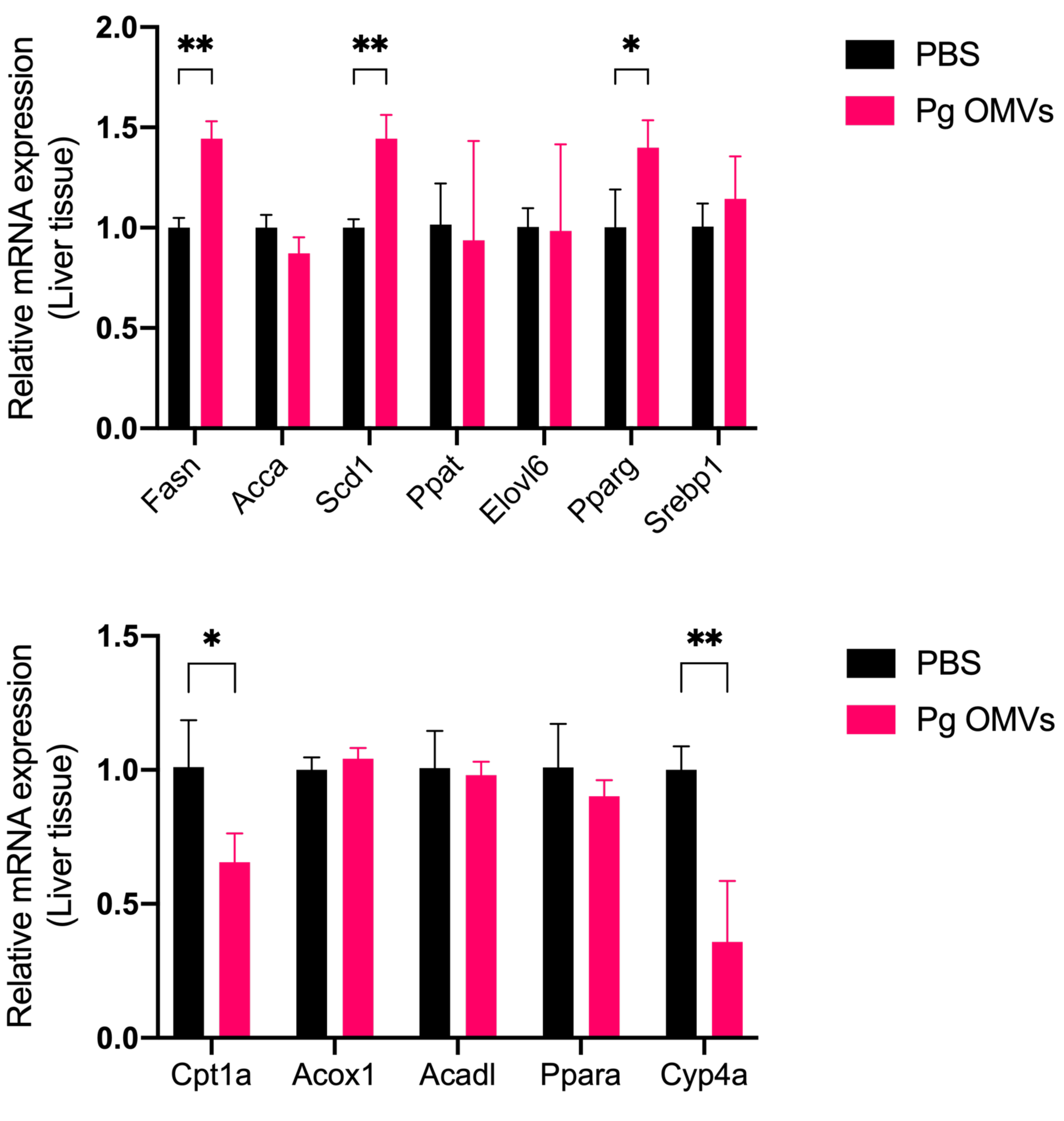

Fig. S2 mRNA expression of genes related to fat metabolism in liver of fat mice after *P. gingivalis* OMVs stimulation.

a mRNA expression of genes related to fat synthesis.

b mRNA expression of genes related to fat oxidation.

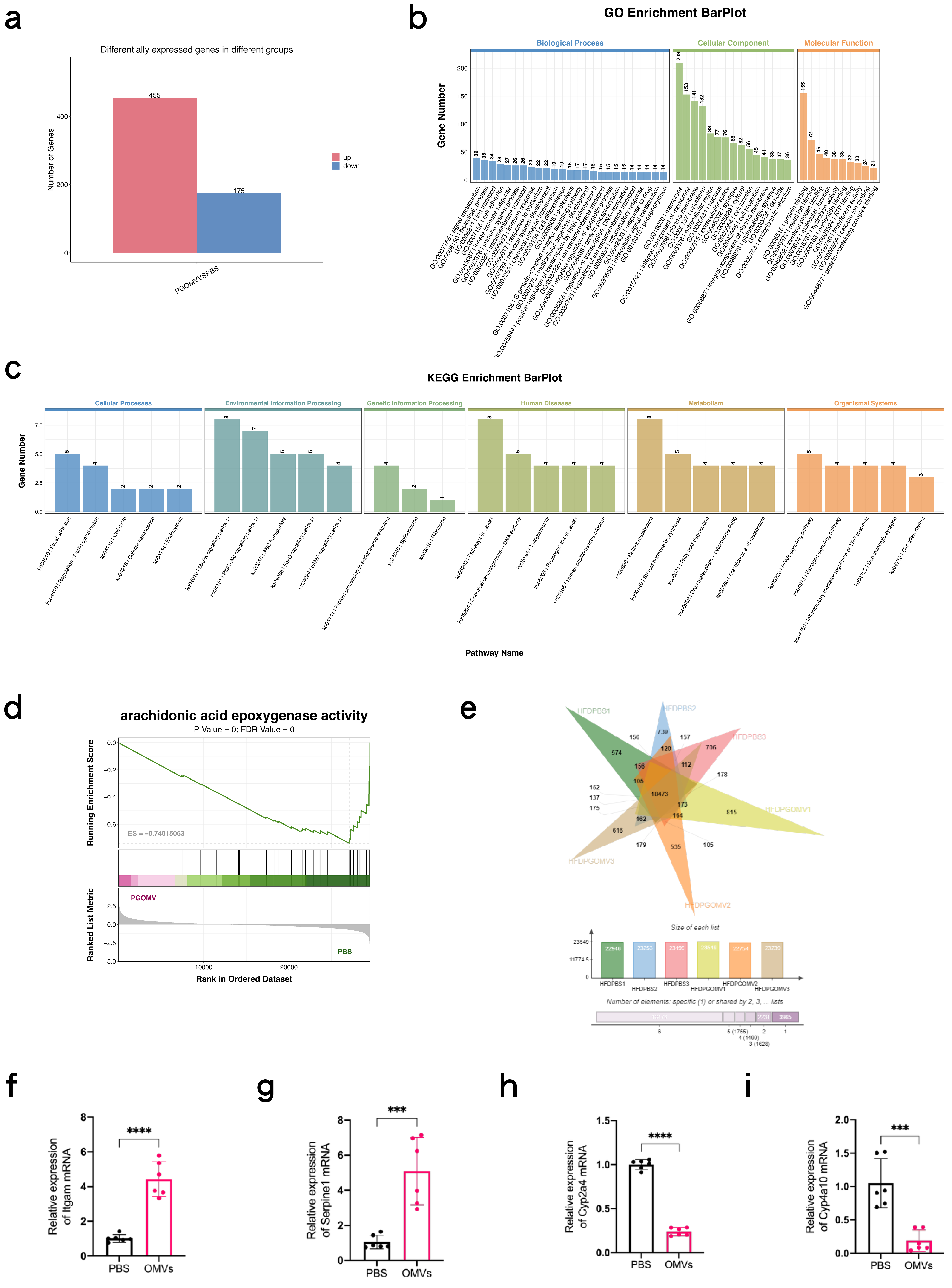

Fig. S3 Transcriptome sequencing on liver tissues from fat mice after *P. gingivalis* OMVs stimulation. **a** The barplot of differentially expressed genes. **b** GO enrichment barplot. **c** KEGG enrichment barplot. **d** ES line diagram for arachidonic acid epoxygenase activity. **e** Venn plot. **f-i** mRNA expression of Itgam, Serpine1, Cyp2a4 and Cyp4a10.

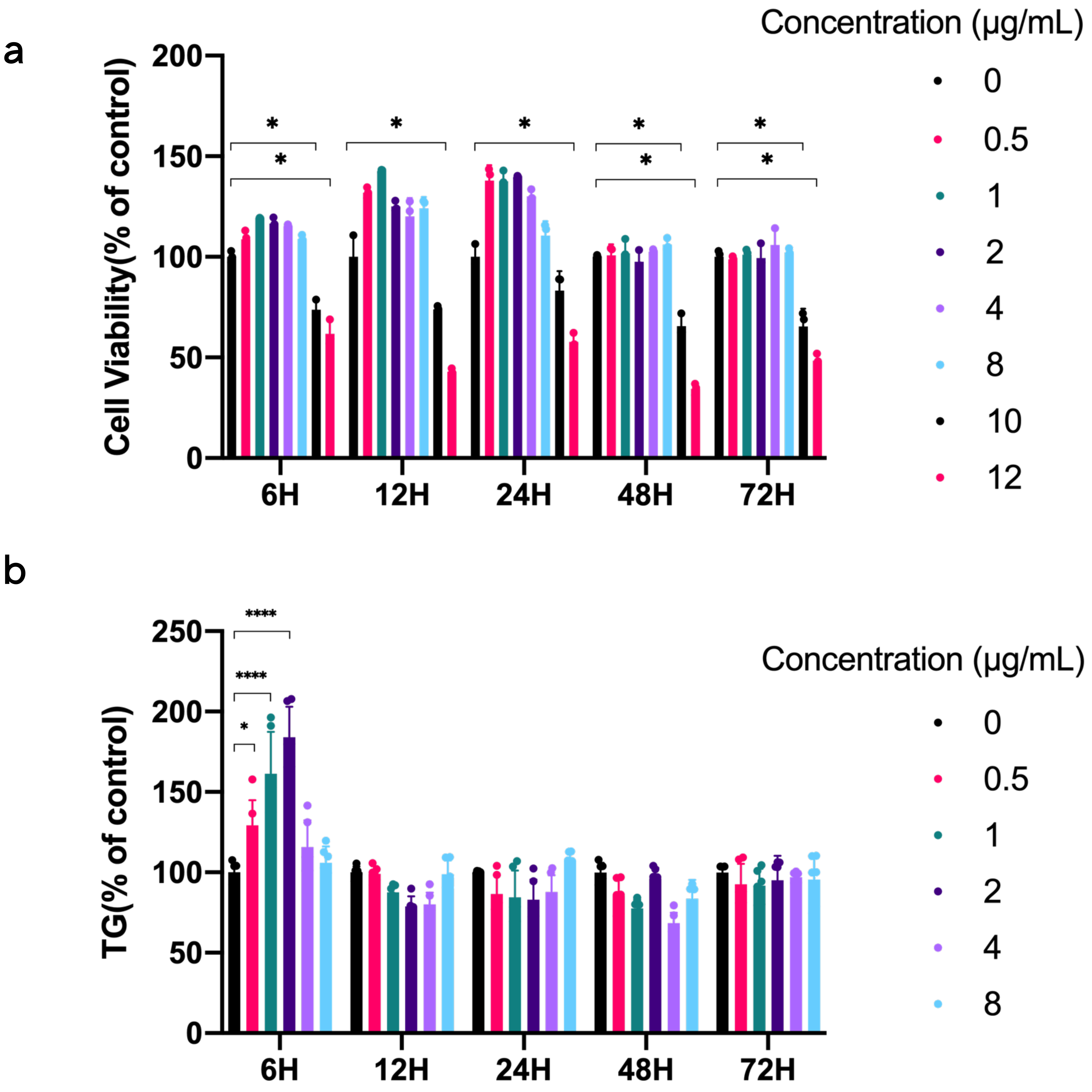

Fig. S4 The activity and triglyceride content of AML12 cells were measured when omv was administered at different time and with different concentration.

a The comparison of cell viability. **b** The comparison of triglyceride content.

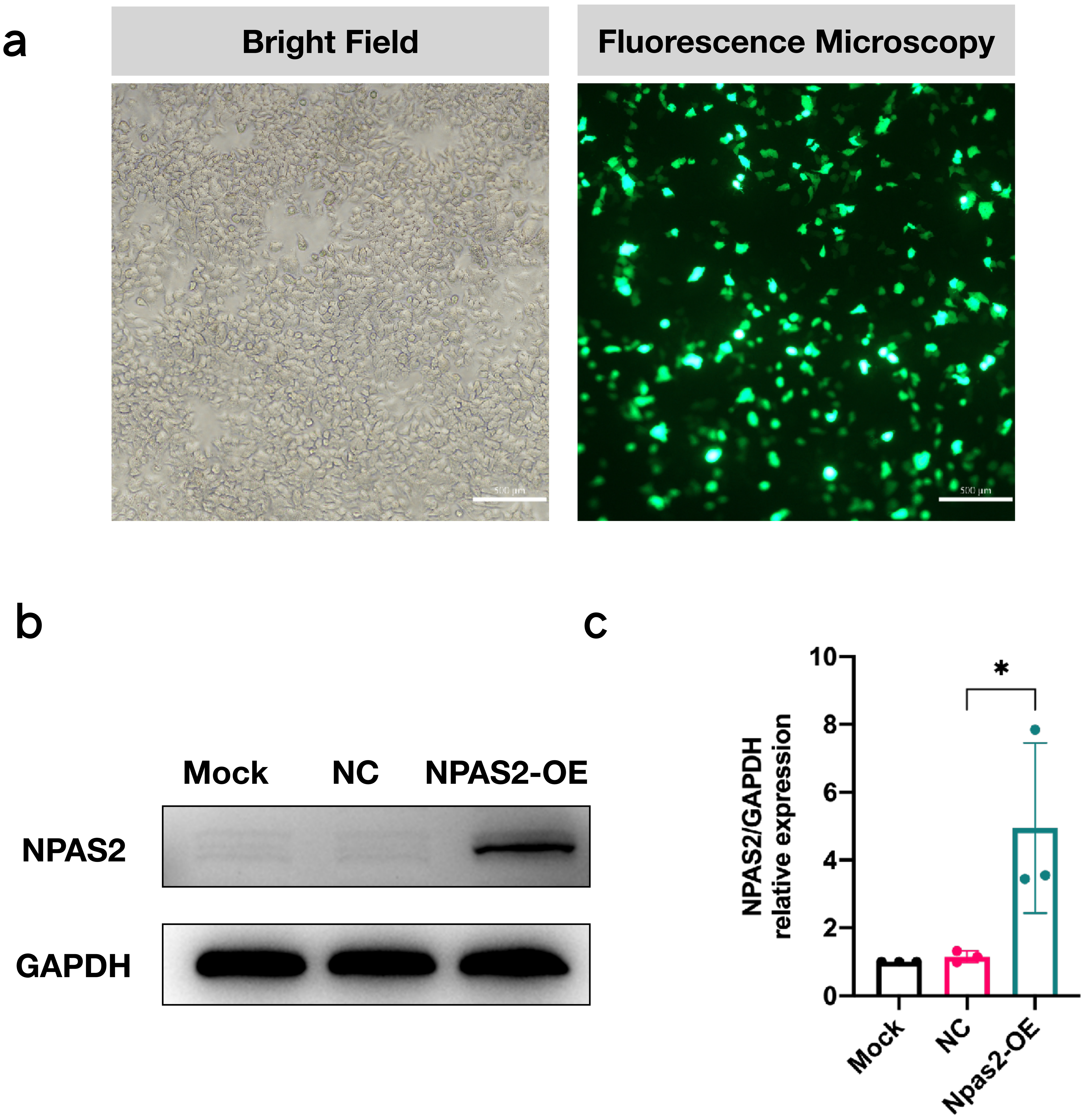

Fig. S5 Fluorescence microscopy and western blot verified that Npas2 was successfully overexpressed.

a Efficiency of successfully transfected AML12 cells under fluorescence microscopy.

b, c Western blot verified the overexpression of NPAS2 in AML12 cells after successful transfection. NC: Negative Control, OE: Over-Expression, $*P<0.05$

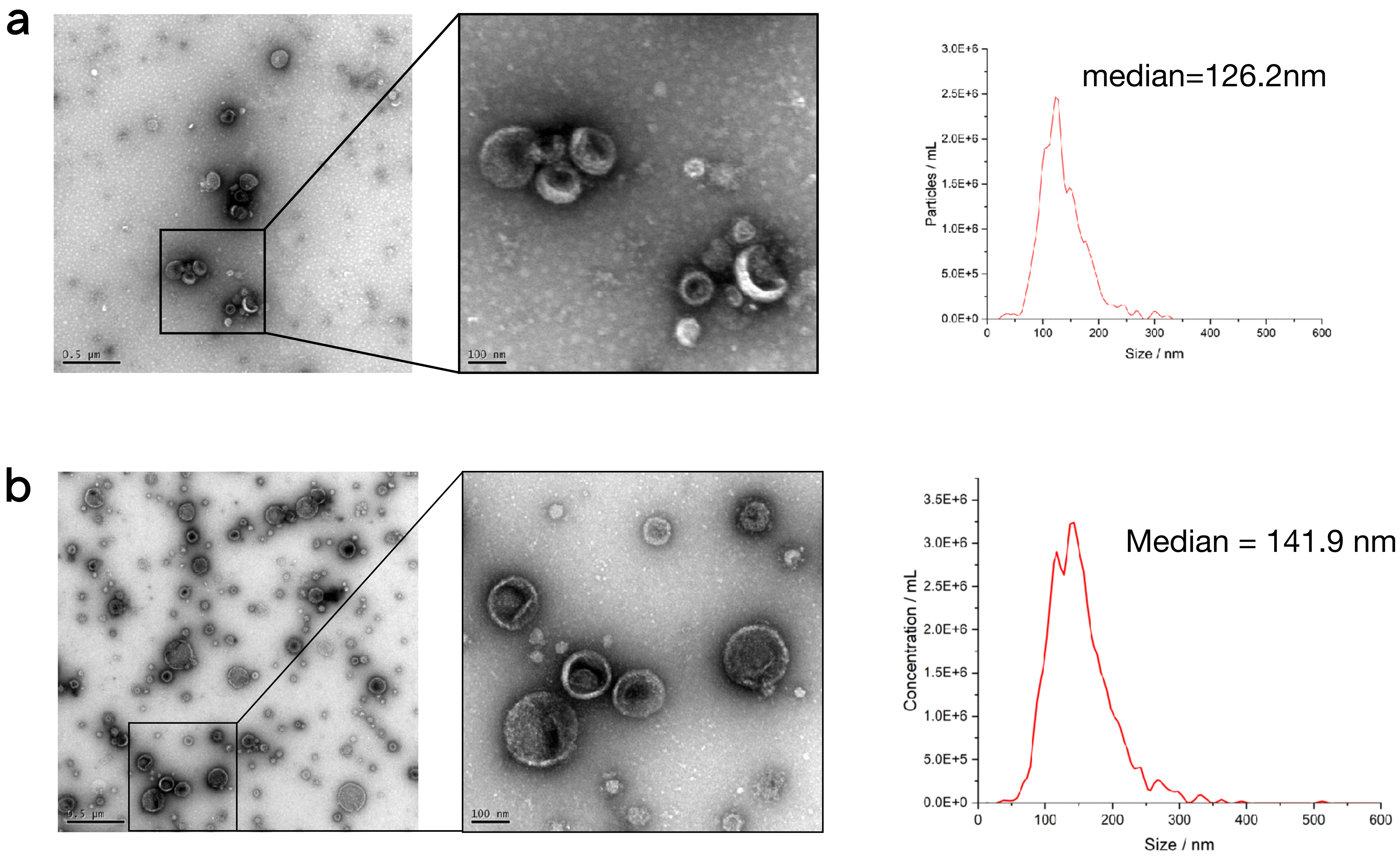

Fig. S6 *P. gingivalis*$^{\Delta ppad}$ and *P. gingivalis*$^{ppad\text{-}OE}$ OMVs were extracted and analyzed through transmission electron microscopy (TEM) and Nanoparticle tracking analysis (NTA).

a TEM showed the *P. gingivalis*$^{\Delta ppad}$ OMVs were vesicle-like structure, NTA showed the diameter of *P. gingivalis*$^{\Delta ppad}$ OMVs is 126.2 nm.

b TEM showed the *P. gingivalis*$^{ppad\text{-}OE}$ OMVs were vesicle-like structure, NTA showed the diameter of *P. gingivalis*$^{\Delta ppad}$ OMVs is 141.9 nm.

a

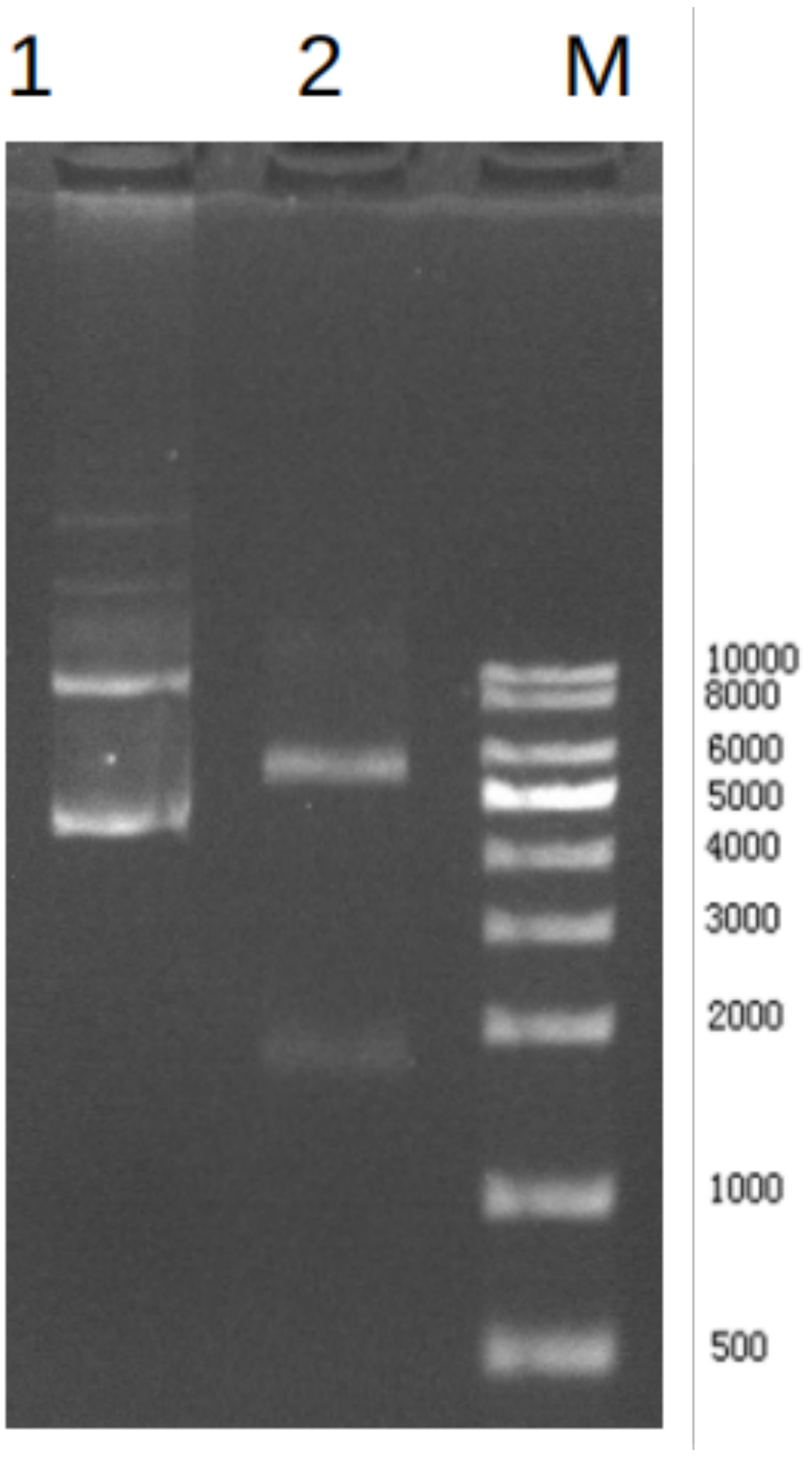

b

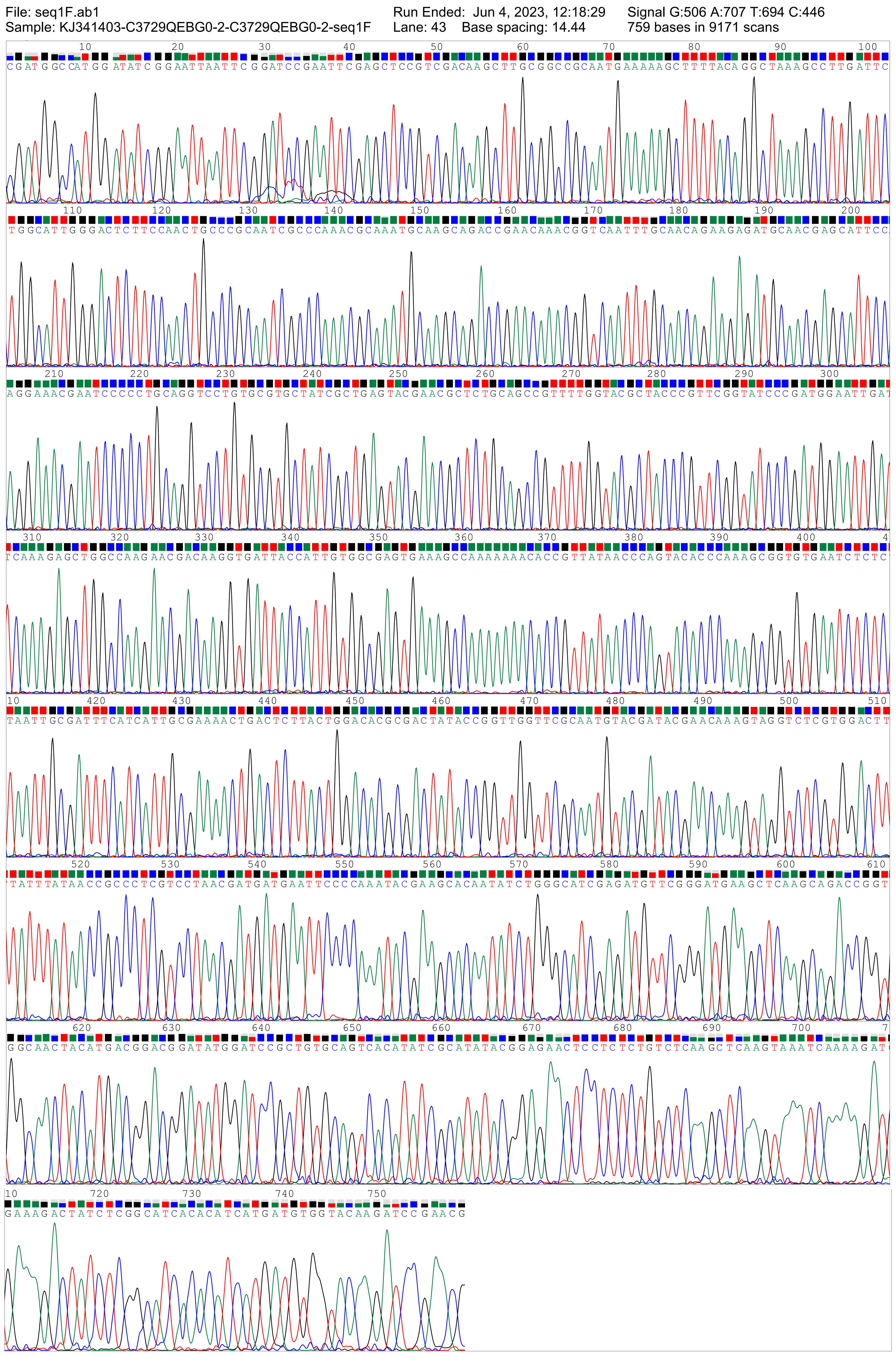

c

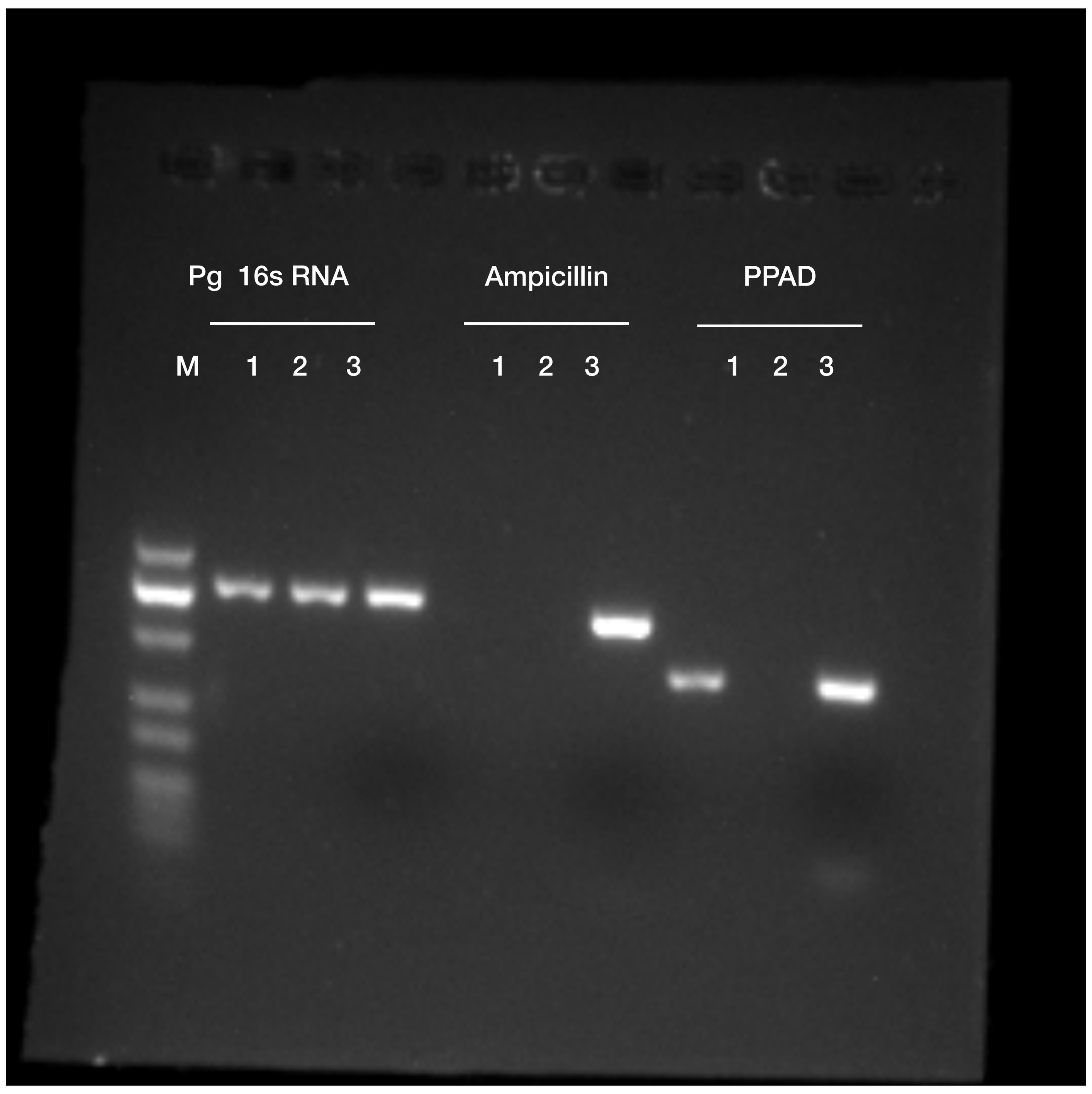

1. *P. gingivalis*
2. *P. gingivalis*$^{\Delta ppad}$
3. *P. gingivalis*$^{ppad\text{-}OE}$

Fig. S7 The PPAD-overexpressing strain *P. gingivalis*$^{ppad\text{-}OE}$ was successfully constructed. **a** Results of plasmid enzyme digestion. Lane M: KB Ladder; Lane 1: pET-22b(+) - PPAD plasmid; Lane 2: pET-22b(+) - PPAD plasmid digested by SacI and XhoI. **b** HIS-pET-22b(+)-PPAD plasmid sequencing results. **c** Agarose gel electrophoresis of DNA for identification of *P. gingivalis*$^{ppad\text{-}OE}$ carrying the ampicillin resistance gene.

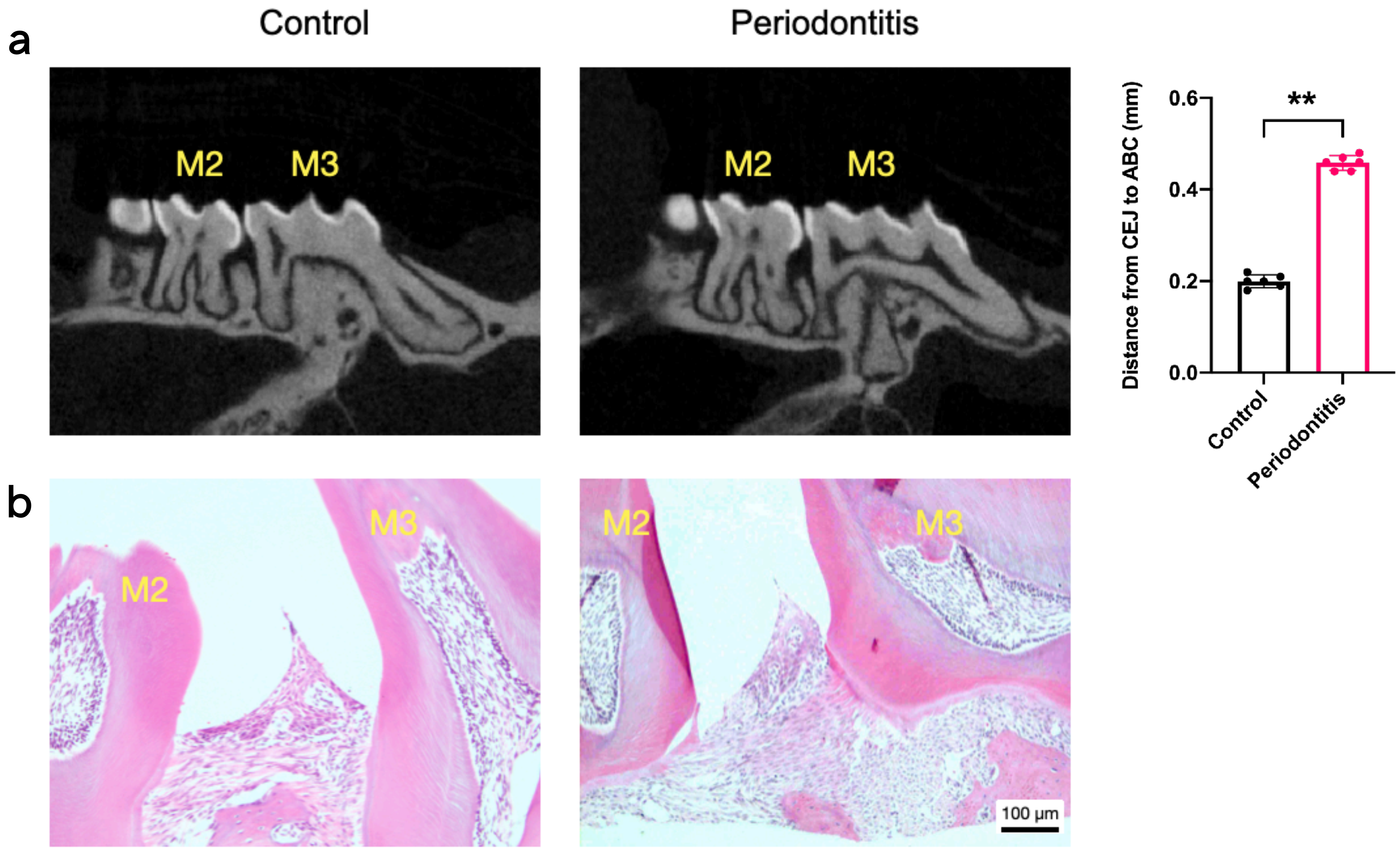

Fig. S8 The successful construction of the periodontitis model in mice

a Micro-CT scan of the maxillary molars and alveolar bone images of mice; Statistical chart of alveolar bone resorption volume **b** H&E staining. Control: Control group, Periodontitis: Periodontitis group, CEJ: Cementum enamel, ABC: Alveolar crest, M2: Second molar, M3: Third molar. n = 6, scale = 100 μm, **, $P < 0.01$.
